# Supplementary material for: Application of CCTV Methodology to Analyze COVID-19 Evolution in Italy
Source: BioTech (Basel). 2022 Aug 11;11(3):33. doi: 10.3390/biotech11030033 (PMC9460631; doi:10.3390/biotech11030033)
Supplement: Supplementary file 1 [file biotech-11-00033-s001.zip › biotech-1814902-SI.pdf]

BioTech

Article

Application of CCTV Methodology to Analyze COVID-19 Evolution in Italy

Marianna Milano [1,*], Giuseppe Agapito [2] and Mario Cannataro [1]

[1] Department of Medical and Surgical Sciences, Data Analytics Research Center, University Magna Græcia, 88100 Catanzaro, Italy
[2] Department of Law, Economics and Social Sciences, University Magna Græcia of Catanzaro, 88100 Catanzaro, Italy
* Correspondence: m.milano@unicz.it

Abstract: Italy was one of the European countries most afflicted by the COVID-19 pandemic. From 2020 to 2022, Italy adopted strong containment measures against the COVID-19 epidemic and then started an important vaccination campaign. Here, we extended previous work by applying the COVID-19 Community Temporal Visualizer (CCTV) methodology to Italian COVID-19 data related to 2020, 2021, and five months of 2022. The aim of this work was to evaluate how Italy reacted to the pandemic in the first two waves of COVID-19, in which only containment measures such as the lockdown had been adopted, in the months following the start of the vaccination campaign, the months with the mildest weather, and the months affected by the new COVID-19 variants. This assessment was conducted by observing the behavior of single regions. CCTV methodology allows us to map the similarities in the behavior of Italian regions on a graph and use a community detection algorithm to visualize and analyze the spatio-temporal evolution of data. The results depict that the communities formed by Italian regions change with respect to the ten data measures and time.

Keywords: COVID-19; CCTV methodology; network analysis; community detection

check for updates

Citation: Milano, M.; Agapito, G.; Cannataro, M. Application of CCTV Methodology to Analyze COVID-19 Evolution in Italy. *BioTech* **2022**, *11*, 33. https://doi.org/10.3390/biotech11030033

Academic Editor: Luca Bianco

Received: 28 June 2022
Accepted: 2 August 2022
Published: 11 August 2022

Publisher's Note: MDPI stays neutral with regard to jurisdictional claims in published maps and institutional affiliations.

Copyright: © 2022 by the authors. Licensee MDPI, Basel, Switzerland. This article is an open access article distributed under the terms and conditions of the Creative Commons Attribution (CC BY) license (https://creativecommons.org/licenses/by/4.0/).

1. Statistical analysis

In this section we reported the results obtained by applying Wilcoxon Sum Rank test. We present different tables that report the results of Wilcoxon Sum Rank test related to Italian COVID-19 data in five temporal intervals: in the first period, second period, third period, fourth period, fifth period. The Tables report the results only with $p - values >= 0.05$. The $p - values < 0.05$ are mapped to zero.

Tables S1–S5, report the similarity matrix related to Hospitalised with Symptoms network in the first period, second period, third period, fourth period, fifth period.

Tables S6–S10, report the similarity matrix related to Intensive Care network in the first period, second period, third period, fourth period, fifth period.

Tables S11–S15, report the similarity matrix related to Total Hospitalised network in the first period, second period, third period, fourth period, fifth period.

Tables S16–S20, report the similarity matrix related to Home Isolation network in the first period, second period, third period, fourth period, fifth period.

Tables S21–S25, report the similarity matrix related to Total Currently Positive network in the first period, second period, third period, fourth period, fifth period.

Tables S26–S30, report the similarity matrix related to New Currently Positive network iin the first period, second period, third period, fourth period, fifth period.

Tables S31–S35, report the similarity matrix related to Discharged/ Healed network in the first period, second period, third period, fourth period, fifth period.

Tables S36–S40, report the similarity matrix related to Deceased network in the first period, second period, third period, fourth period, fifth period.

Tables S41–S45, report the similarity matrix related to Total Cases in the first period, second period, third period, fourth period, fifth period.

Tables S46–S50, report the similarity matrix related to Swabs in the first period, second period, third period, fourth period, fifth period.

Table S1. Similarity Matrix of Hospitalised with Symptoms network in the first period.

	Abruzzo	Basilicata	Bolzano	Calabria	Campania	Emilia	Friuli	Lazio	Liguria	Lombardia	Marche	Molise	Piemonte	Puglia	Sardegna	Sicilia	Toscana	Trento	Umbria	ValleAosta	Veneto
Abruzzo	1	0	0	0	0	0	0	0	0	0	0	0	0	0	0	0	0	0	0	0	0
Basilicata	0	1	0	0	0	0	0	0	0	0	0	0	0	0	0	0	0	0	0	0	0
Bolzano	0	0	1	0.1	0	0	0.6	0	0	0	0	0	0	0	0	0	0	0.05	0	0	0
Calabria	0	0	0.1	1	0	0	0	0	0	0	0	0	0	0	0.28	0	0	0	0.13	0	0
Campania	0	0	0	0	1	0	0	0	0	0	0	0	0	0.66	0	0	0	0	0	0	0
Emilia	0	0	0	0	0	1	0	0	0	0	0	0	0.97	0	0	0	0	0	0	0	0
Friuli	0	0	0.6	0	0	0	1	0	0	0	0	0	0	0	0	0	0	0.12	0	0	0
Lazio	0	0	0	0	0	0	0	1	0	0	0	0	0	0	0	0	0	0	0	0	0.39
Liguria	0	0	0	0	0	0	0	0	1	0	0.21	0	0	0	0	0	0.38	0	0	0	0
Lombardia	0	0	0	0	0	0	0	0	0	1	0	0	0	0	0	0	0	0	0	0	0
Marche	0	0	0	0	0	0	0	0	0.21	0	1	0	0	0	0	0	0.5	0	0	0	0
Molise	0	0	0	0	0	0	0	0	0	0	0	1	0	0	0	0	0	0	0	0	0
Piemonte	0	0	0	0	0	0.97	0	0	0	0	0	0	1	0	0	0	0	0	0	0	0
Puglia	0	0	0	0	0.66	0	0	0	0	0	0	0	0	1	0	0	0	0	0	0	0
Sardegna	0	0	0	0.28	0	0	0	0	0	0	0	0	0	0	1	0	0	0	0.64	0	0
Sicilia	0	0	0	0	0	0	0	0	0	0	0	0	0	0	0	1	0	0	0	0	0
Toscana	0	0	0	0	0	0	0	0	0.38	0	0.5	0	0	0	0	0	1	0	0	0	0
Trento	0	0	0.05	0	0	0	0.12	0	0	0	0	0	0	0	0	0	0	1	0	0	0
Umbria	0	0	0	0.13	0	0	0	0	0	0	0	0	0	0	0.64	0	0	0	1	0.18	0
ValleAosta	0	0	0	0	0	0	0	0	0	0	0	0	0	0	0	0	0	0	0.18	1	0
Veneto	0	0	0	0	0	0	0	0.39	0	0	0	0	0	0	0	0	0	0	0	0	1

Table S2. Similarity Matrix of Hospitalised with Symptoms network in the second period.

	Abruzzo	Basilicata	Bolzano	Calabria	Campania	Emilia	Friuli	Lazio	Liguria	Lombardia	Marche	Molise	Piemonte	Puglia	Sardegna	Sicilia	Toscana	Trento	Umbria	ValleAosta	Veneto
Abruzzo	1	0	0	0	0	0	0	0	0	0	0	0	0	0	0.06	0	0	0	0	0	0
Basilicata	0	1	0	0	0	0	0	0	0	0	0	0	0	0	0	0	0	0	0	0.59	0
Bolzano	0	0	1	0.56	0	0	0	0	0	0	0	0	0	0	0	0	0	0.49	0.89	0	0
Calabria	0	0	0.56	1	0	0	0	0	0	0	0	0	0	0	0	0	0	0.35	0.9	0	0
Campania	0	0	0	0	1	0	0	0	0	0	0	0	0	0	0	0	0	0	0	0	0.12
Emilia	0	0	0	0	0	1	0	0	0	0	0	0	0	0	0	0	0	0	0	0	0
Friuli	0	0	0	0	0	0	1	0	0	0	0.16	0	0	0	0.92	0	0	0	0	0	0
Lazio	0	0	0	0	0	0	0	1	0	0	0	0	0	0	0	0	0	0	0	0	0
Liguria	0	0	0	0	0	0	0	0	1	0	0	0	0	0	0	0	0	0	0	0	0
Lombardia	0	0	0	0	0	0	0	0	0	1	0	0	0	0	0	0	0	0	0	0	0
Marche	0	0	0	0	0	0	0.16	0	0	0	1	0	0	0	0.06	0	0	0	0	0	0
Molise	0	0	0	0	0	0	0	0	0	0	0	1	0	0	0	0	0	0	0	0	0
Piemonte	0	0	0	0	0	0	0	0	0	0	0	0	1	0	0	0	0	0	0	0	0
Puglia	0	0	0	0	0	0	0	0	0	0	0	0	0	1	0	0.41	0.72	0	0	0	0
Sardegna	0.06	0	0	0	0	0	0.92	0	0	0	0.06	0	0	0	1	0	0	0	0	0	0
Sicilia	0	0	0	0	0	0	0	0	0	0	0	0	0	0.41	0	1	0.96	0	0	0	0
Toscana	0	0	0	0	0	0	0	0	0	0	0	0	0	0.72	0	0.96	1	0	0	0	0
Trento	0	0	0.49	0.35	0	0	0	0	0	0	0	0	0	0	0	0	0	1	0.07	0	0
Umbria	0	0	0.89	0.9	0	0	0	0	0	0	0	0	0	0	0	0	0	0.07	1	0	0
ValleAosta	0	0.59	0	0	0	0	0	0	0	0	0	0	0	0	0	0	0	0	0	1	0
Veneto	0	0	0	0	0.12	0	0	0	0	0	0	0	0	0	0	0	0	0	0	0	1

Table S3. Similarity Matrix of Hospitalised with Symptoms network in the third period.

	Abruzzo	Basilicata	Bolzano	Calabria	Campania	Emilia	Friuli	Lazio	Liguria	Lombardia	Marche	Molise	Piemonte	Puglia	Sardegna	Sicilia	Toscana	Trento	Umbria	ValleAosta	Veneto
Abruzzo	1	0	0	0	0	0	0.44	0	0	0	0	0	0	0	0	0	0	0	0	0	0
Basilicata	0	1	0.06	0	0	0	0	0	0	0	0	0	0	0	0	0	0	0	0	0	0
Bolzano	0	0.06	1	0	0	0	0	0	0	0	0	0	0	0	0	0	0	0.27	0	0	0
Calabria	0	0	0	1	0	0	0	0	0	0	0	0	0	0	0.47	0	0	0	0.24	0	0
Campania	0	0	0	0	1	0	0	0	0	0	0	0	0	0.48	0	0	0	0	0	0	0
Emilia	0	0	0	0	0	1	0	0.66	0	0	0	0	0.31	0	0	0	0	0	0	0	0
Friuli	0.44	0	0	0	0	0	1	0	0	0	0	0	0	0	0	0	0	0	0	0	0
Lazio	0	0	0	0	0	0.66	0	1	0	0	0	0	0.69	0	0	0	0	0	0	0	0
Liguria	0	0	0	0	0	0	0	0	1	0	0.5	0	0	0	0	0	0	0	0	0	0
Lombardia	0	0	0	0	0	0	0	0	0	1	0	0	0	0	0	0	0	0	0	0	0
Marche	0	0	0	0	0	0	0	0	0.5	0	1	0	0	0	0	0	0	0	0	0	0
Molise	0	0	0	0	0	0	0	0	0	0	0	1	0	0	0	0	0	0	0	0	0
Piemonte	0	0	0	0	0	0.31	0	0.69	0	0	0	0	1	0	0	0	0	0	0	0	0
Puglia	0	0	0	0	0.48	0	0	0	0	0	0	0	0	1	0	0	0	0	0	0	0
Sardegna	0	0	0	0.47	0	0	0	0	0	0	0	0	0	0	1	0	0	0	0.25	0	0
Sicilia	0	0	0	0	0	0	0	0	0	0	0	0	0	0	0	1	0.72	0	0	0	0
Toscana	0	0	0	0	0	0	0	0	0	0	0	0	0	0	0	0.72	1	0	0	0	0
Trento	0	0	0.27	0	0	0	0	0	0	0	0	0	0	0	0	0	0	1	0	0	0
Umbria	0	0	0	0.24	0	0	0	0	0	0	0	0	0	0	0.25	0	0	0	1	0	0
ValleAosta	0	0	0	0	0	0	0	0	0	0	0	0	0	0	0	0	0	0	0	1	0
Veneto	0	0	0	0	0	0	0	0	0	0	0	0	0	0	0	0	0	0	0	0	1

Table S4. Similarity Matrix of Hospitalised with Symptoms network in the fourth period.

	Abruzzo	Basilicata	Bolzano	Calabria	Campania	Emilia	Friuli	Lazio	Liguria	Lombardia	Marche	Molise	Piemonte	Puglia	Sardegna	Sicilia	Toscana	Trento	Umbria	ValleAosta	Veneto
Abruzzo	1	0	0	0	0	0	0	0	0.65	0	0.23	0	0	0	0	0	0	0	0	0	0
Basilicata	0	1	0	0	0	0	0	0	0	0	0	0	0	0	0	0	0	0	0.25	0	0
Bolzano	0	0	1	0	0	0	0.06	0	0	0	0	0	0	0	0	0	0	0.58	0	0	0
Calabria	0	0	0	1	0	0	0	0	0	0	0	0	0	0	0.23	0	0	0	0	0	0.06
Campania	0	0	0	0	1	0.18	0	0	0	0	0	0	0	0	0	0	0	0	0	0	0
Emilia	0	0	0	0	0.18	1	0	0	0	0	0	0	0	0	0	0	0	0	0	0	0
Friuli	0	0	0.06	0	0	0	1	0	0	0	0	0	0	0	0	0	0	0	0	0	0
Lazio	0	0	0	0	0	0	0	1	0	0.3	0	0	0	0	0	0.35	0	0	0	0	0
Liguria	0.65	0	0	0	0	0	0	0	1	0	0	0	0	0	0	0	0	0	0	0	0
Lombardia	0	0	0	0	0	0	0	0.3	0	1	0	0	0	0	0	0.24	0	0	0	0	0
Marche	0.23	0	0	0	0	0	0	0	0	0	1	0	0	0	0	0	0	0	0	0	0
Molise	0	0	0	0	0	0	0	0	0	0	0	1	0	0	0	0	0	0	0	0	0
Piemonte	0	0	0	0	0	0	0	0	0	0	0	0	1	0.73	0	0	0	0	0	0	0
Puglia	0	0	0	0	0	0	0	0	0	0	0	0	0.73	1	0	0	0	0	0	0	0
Sardegna	0	0	0	0.23	0	0	0	0	0	0	0	0	0	0	1	0	0	0	0	0	0.4
Sicilia	0	0	0	0	0	0	0	0.35	0	0.24	0	0	0	0	0	1	0	0	0	0	0
Toscana	0	0	0	0	0	0	0	0	0	0	0	0	0	0	0	0	1	0	0	0	0
Trento	0	0	0.58	0	0	0	0	0	0	0	0	0	0	0	0	0	0	1	0	0	0
Umbria	0	0.25	0	0	0	0	0	0	0	0	0	0	0	0	0	0	0	0	1	0	0
ValleAosta	0	0	0	0	0	0	0	0	0	0	0	0	0	0	0	0	0	0	0	1	0
Veneto	0	0	0	0.06	0	0	0	0	0	0	0	0	0	0	0.4	0	0	0	0	0	1

Table S5. Similarity Matrix of Hospitalised with Symptoms network in the fifth period.

	Abruzzo	Basilicata	Bolzano	Calabria	Campania	Emilia	Friuli	Lazio	Liguria	Lombardia	Marche	Molise	Piemonte	Puglia	Sardegna	Sicilia	Toscana	Trento	Umbria	ValleAosta	Veneto
Abruzzo	1	0	0.11	0	0	0	0.9	0	0	0	0	0	0	0	0.22	0	0	0.06	0	0	0
Basilicata	0	1	0	0	0	0	0	0	0	0	0	0	0	0	0	0	0	0	0	0	0
Bolzano	0.11	0	1	0	0	0	0	0	0	0	0	0	0	0	0	0	0	0.51	0	0	0
Calabria	0	0	0	1	0	0	0.18	0	0	0	0	0	0	0	0	0	0	0	0	0	0
Campania	0	0	0	0	1	0	0	0	0	0	0	0	0	0	0	0	0.17	0	0	0	0
Emilia	0	0	0	0	0	1	0	0	0	0	0	0	0.8	0	0	0	0	0	0	0	0
Friuli	0.9	0	0	0.18	0	0	1	0	0	0	0	0	0	0	0.57	0	0	0	0	0	0
Lazio	0	0	0	0	0	0	0	1	0	0.13	0	0	0.21	0	0	0	0	0	0	0	0
Liguria	0	0	0	0	0	0	0	0	1	0	0	0	0	0.06	0	0	0	0	0	0	0
Lombardia	0	0	0	0	0	0	0	0.13	0	1	0	0	0	0	0	0	0	0	0	0	0
Marche	0	0	0	0	0	0	0	0	0	0	1	0	0	0	0	0	0	0	0	0	0
Molise	0	0	0	0	0	0	0	0	0	0	0	1	0	0	0	0	0	0	0	0	0
Piemonte	0	0	0	0	0	0.8	0	0.21	0	0	0	0	1	0	0	0	0	0	0	0	0
Puglia	0	0	0	0	0	0	0	0	0.06	0	0	0	0	1	0	0	0	0	0	0	0
Sardegna	0.22	0	0	0	0	0	0.57	0	0	0	0	0	0	0	1	0	0	0	0	0	0
Sicilia	0	0	0	0	0	0	0	0	0	0	0	0	0	0	0	1	0	0	0	0	0
Toscana	0	0	0	0	0.17	0	0	0	0	0	0	0	0	0	0	0	1	0	0	0	0
Trento	0.06	0	0.51	0	0	0	0	0	0	0	0	0	0	0	0	0	0	1	0.6	0	0
Umbria	0	0	0	0	0	0	0	0	0	0	0	0	0	0	0	0	0	0.6	1	0	0
ValleAosta	0	0	0	0	0	0	0	0	0	0	0	0	0	0	0	0	0	0	0	1	0
Veneto	0	0	0	0	0	0	0	0	0	0	0	0	0	0	0	0	0	0	0	0	1

Table S6. Similarity Matrix of Intensive Care network in the first period.

	Abruzzo	Basilicata	Bolzano	Calabria	Campania	Emilia	Friuli	Lazio	Liguria	Lombardia	Marche	Molise	Piemonte	Puglia	Sardegna	Sicilia	Toscana	Trento	Umbria	ValleAosta	Veneto
Abruzzo	1	0	0.12	0	0	0	0	0	0	0	0	0	0	0	0	0.25	0	0.87	0	0	0
Basilicata	0	1	0	0.17	0	0	0	0	0	0	0	0.16	0	0	0	0	0	0	0	0.65	0
Bolzano	0.12	0	1	0	0	0	0.24	0	0	0	0	0	0	0	0.17	0	0	0.11	0.61	0	0
Calabria	0	0.17	0	1	0	0	0	0	0	0	0	0	0	0	0	0	0	0	0	0.49	0
Campania	0	0	0	0	1	0	0	0	0	0	0	0	0	0.99	0	0.1	0	0	0	0	0
Emilia	0	0	0	0	0	1	0	0	0	0	0	0	0.79	0	0	0	0	0	0	0	0
Friuli	0	0	0.24	0	0	0	1	0	0	0	0	0	0	0	0.27	0	0	0	0.58	0	0
Lazio	0	0	0	0	0	0	0	1	0	0	0	0	0	0	0	0	0.06	0	0	0	0.09
Liguria	0	0	0	0	0	0	0	0	1	0	0.7	0	0	0	0	0	0	0	0	0	0
Lombardia	0	0	0	0	0	0	0	0	0	1	0	0	0	0	0	0	0	0	0	0	0
Marche	0	0	0	0	0	0	0	0	0.7	0	1	0	0	0	0	0	0	0	0	0	0
Molise	0	0.16	0	0	0	0	0	0	0	0	0	1	0	0	0	0	0	0	0	0.11	0
Piemonte	0	0	0	0	0	0.79	0	0	0	0	0	0	1	0	0	0	0	0	0	0	0
Puglia	0	0	0	0	0.99	0	0	0	0	0	0	0	0	1	0	0.06	0	0	0	0	0
Sardegna	0	0	0.17	0	0	0	0.27	0	0	0	0	0	0	0	1	0	0	0	0.07	0	0
Sicilia	0.25	0	0	0	0.1	0	0	0	0	0	0	0	0	0.06	0	1	0	0.36	0	0	0
Toscana	0	0	0	0	0	0	0	0.06	0	0	0	0	0	0	0	0	1	0	0	0	0.87
Trento	0.87	0	0.11	0	0	0	0	0	0	0	0	0	0	0	0	0.36	0	1	0	0	0
Umbria	0	0	0.61	0	0	0	0.58	0	0	0	0	0	0	0	0.07	0	0	0	1	0	0
ValleAosta	0	0.65	0	0.49	0	0	0	0	0	0	0	0.11	0	0	0	0	0	0	0	1	0
Veneto	0	0	0	0	0	0	0	0.09	0	0	0	0	0	0	0	0	0.87	0	0	0	1

Table S7. Similarity Matrix of Intensive Care network in the second period.

	Abruzzo	Basilicata	Bolzano	Calabria	Campania	Emilia	Friuli	Lazio	Liguria	Lombardia	Marche	Molise	Piemonte	Puglia	Sardegna	Sicilia	Toscana	Trento	Umbria	ValleAosta	Veneto
Abruzzo	1	0	0	0	0	0	0.44	0	0	0	0	0	0	0	0.12	0	0	0	0.86	0	0
Basilicata	0	1	0	0	0	0	0	0	0	0	0	0	0	0	0	0	0	0	0	0	0
Bolzano	0	0	1	0.19	0	0	0	0	0	0	0	0	0	0	0	0	0	0	0	0	0
Calabria	0	0	0.19	1	0	0	0	0	0	0	0	0	0	0	0	0	0	0.06	0	0	0
Campania	0	0	0	0	1	0	0	0	0	0	0	0	0	0.85	0	0	0	0	0	0	0
Emilia	0	0	0	0	0	1	0	0	0	0	0	0	0	0	0	0	0.32	0	0	0	0
Friuli	0.44	0	0	0	0	0	1	0	0	0	0	0	0	0	0	0	0	0	0.47	0	0
Lazio	0	0	0	0	0	0	0	1	0	0	0	0	0.65	0	0	0	0	0	0	0	0.16
Liguria	0	0	0	0	0	0	0	0	1	0	0	0	0	0	0	0	0	0	0	0	0
Lombardia	0	0	0	0	0	0	0	0	0	1	0	0	0	0	0	0	0	0	0	0	0
Marche	0	0	0	0	0	0	0	0	0	0	1	0	0	0	0	0	0	0	0	0	0
Molise	0	0	0	0	0	0	0	0	0	0	0	1	0	0	0	0	0	0	0	0	0
Piemonte	0	0	0	0	0	0	0	0.65	0	0	0	0	1	0	0	0	0	0	0	0	0.4
Puglia	0	0	0	0	0.85	0	0	0	0	0	0	0	0	1	0	0	0	0	0	0	0
Sardegna	0.12	0	0	0	0	0	0	0	0	0	0	0	0	0	1	0	0	0	0.2	0	0
Sicilia	0	0	0	0	0	0	0	0	0	0	0	0	0	0	0	1	0	0	0	0	0
Toscana	0	0	0	0	0	0.32	0	0	0	0	0	0	0	0	0	0	1	0	0	0	0
Trento	0	0	0	0.06	0	0	0	0	0	0	0	0	0	0	0	0	0	1	0	0	0
Umbria	0.86	0	0	0	0	0	0.47	0	0	0	0	0	0	0	0.2	0	0	0	1	0	0
ValleAosta	0	0	0	0	0	0	0	0	0	0	0	0	0	0	0	0	0	0	0	1	0
Veneto	0	0	0	0	0	0	0	0.16	0	0	0	0	0.4	0	0	0	0	0	0	0	1

Table S8. Similarity Matrix of Intensive Care network in the third period.

	Abruzzo	Basilicata	Bolzano	Calabria	Campania	Emilia	Friuli	Lazio	Liguria	Lombardia	Marche	Molise	Piemonte	Puglia	Sardegna	Sicilia	Toscana	Trento	Umbria	ValleAosta	Veneto
Abruzzo	1	0	0	0	0	0	0.36	0	0	0	0	0	0	0	0	0	0	0	0.53	0	0
Basilicata	0	1	0	0	0	0	0	0	0	0	0	0	0	0	0	0	0	0	0	0	0
Bolzano	0	0	1	0	0	0	0	0	0	0	0	0	0	0	0	0	0	0	0	0	0
Calabria	0	0	0	1	0	0	0	0	0	0	0	0	0	0	0	0	0	0	0	0	0
Campania	0	0	0	0	1	0	0	0	0	0	0	0	0	0	0	0	0	0	0	0	0
Emilia	0	0	0	0	0	1	0	0	0	0	0	0	0	0	0	0	0	0	0	0	0
Friuli	0.36	0	0	0	0	0	1	0	0	0	0	0	0	0	0	0	0	0	0.08	0	0
Lazio	0	0	0	0	0	0	0	1	0	0	0	0	0	0	0	0	0	0	0	0	0
Liguria	0	0	0	0	0	0	0	0	1	0	0	0	0	0	0	0	0	0	0	0	0
Lombardia	0	0	0	0	0	0	0	0	0	1	0	0	0	0	0	0	0	0	0	0	0
Marche	0	0	0	0	0	0	0	0	0	0	1	0	0	0	0	0	0	0	0	0	0
Molise	0	0	0	0	0	0	0	0	0	0	0	1	0	0	0	0	0	0	0	0	0
Piemonte	0	0	0	0	0	0	0	0	0	0	0	0	1	0.19	0	0	0	0	0	0	0.21
Puglia	0	0	0	0	0	0	0	0	0	0	0	0	0.19	1	0	0	0.92	0	0	0	0.46
Sardegna	0	0	0	0	0	0	0	0	0	0	0	0	0	0	1	0	0	0.11	0	0	0
Sicilia	0	0	0	0	0	0	0	0	0	0	0	0	0	0	0	1	0	0	0	0	0
Toscana	0	0	0	0	0	0	0	0	0	0	0	0	0	0.92	0	0	1	0	0	0	0.47
Trento	0	0	0	0	0	0	0	0	0	0	0	0	0	0	0.11	0	0	1	0	0	0
Umbria	0.53	0	0	0	0	0	0.08	0	0	0	0	0	0	0	0	0	0	0	1	0	0
ValleAosta	0	0	0	0	0	0	0	0	0	0	0	0	0	0	0	0	0	0	0	1	0
Veneto	0	0	0	0	0	0	0	0	0	0	0	0	0.21	0.46	0	0	0.47	0	0	0	1

Table S9. Similarity Matrix of Intensive Care network in the fourth period.

	Abruzzo	Basilicata	Bolzano	Calabria	Campania	Emilia	Friuli	Lazio	Liguria	Lombardia	Marche	Molise	Piemonte	Puglia	Sardegna	Sicilia	Toscana	Trento	Umbria	ValleAosta	Veneto
Abruzzo	1	0	0.42	0	0	0	0.36	0	0	0	0	0	0	0	0	0	0	0	0.09	0	0
Basilicata	0	1	0	0	0	0	0	0	0	0	0	0.16	0	0	0	0	0	0	0	0	0
Bolzano	0.42	0	1	0	0	0	0.21	0	0	0	0	0	0	0	0	0	0	0	0.3	0	0
Calabria	0	0	0	1	0	0	0	0	0	0	0	0	0	0	0	0	0	0	0	0	0
Campania	0	0	0	0	1	0	0	0	0	0	0	0	0.05	0.06	0	0	0	0	0	0	0.69
Emilia	0	0	0	0	0	1	0	0	0	0	0	0	0	0	0	0	0.29	0	0	0	0
Friuli	0.36	0	0.21	0	0	0	1	0	0	0	0	0	0	0	0	0	0	0.08	0	0	0
Lazio	0	0	0	0	0	0	0	1	0	0.42	0	0	0	0	0	0	0	0	0	0	0
Liguria	0	0	0	0	0	0	0	0	1	0	0.82	0	0	0	0.25	0	0	0	0	0	0
Lombardia	0	0	0	0	0	0	0	0.42	0	1	0	0	0	0	0	0	0	0	0	0	0
Marche	0	0	0	0	0	0	0	0	0.82	0	1	0	0	0	0.1	0	0	0	0	0	0
Molise	0	0.16	0	0	0	0	0	0	0	0	0	1	0	0	0	0	0	0	0	0	0
Piemonte	0	0	0	0	0.05	0	0	0	0	0	0	0	1	0.47	0	0	0	0	0	0	0
Puglia	0	0	0	0	0.06	0	0	0	0	0	0	0	0.47	1	0	0	0	0	0	0	0.09
Sardegna	0	0	0	0	0	0	0	0	0.25	0	0.1	0	0	0	1	0	0	0	0	0	0
Sicilia	0	0	0	0	0	0	0	0	0	0	0	0	0	0	0	1	0	0	0	0	0
Toscana	0	0	0	0	0	0.29	0	0	0	0	0	0	0	0	0	0	1	0	0	0	0
Trento	0	0	0	0	0	0	0.08	0	0	0	0	0	0	0	0	0	0	1	0	0	0
Umbria	0.09	0	0.3	0	0	0	0	0	0	0	0	0	0	0	0	0	0	0	1	0	0
ValleAosta	0	0	0	0	0	0	0	0	0	0	0	0	0	0	0	0	0	0	0	1	0
Veneto	0	0	0	0	0.69	0	0	0	0	0	0	0	0	0.09	0	0	0	0	0	0	1

Table S10. Similarity Matrix of Intensive Care network in the fifth period.

	Abruzzo	Basilicata	Bolzano	Calabria	Campania	Emilia	Friuli	Lazio	Liguria	Lombardia	Marche	Molise	Piemonte	Puglia	Sardegna	Sicilia	Toscana	Trento	Umbria	ValleAosta	Veneto
Abruzzo	1	0	0.82	0	0	0	0	0	0	0	0	0	0	0	0	0	0	0.17	0	0	0
Basilicata	0	1	0	0	0	0	0	0	0	0	0	0	0	0	0	0	0	0	0	0.15	0
Bolzano	0.82	0	1	0	0	0	0	0	0	0	0	0	0	0	0	0	0	0	0	0	0
Calabria	0	0	0	1	0	0	0	0	0	0	0	0	0	0	0.47	0	0	0.82	0	0	0
Campania	0	0	0	0	1	0	0	0	0	0	0.85	0	0	0.38	0	0	0	0	0	0	0
Emilia	0	0	0	0	0	1	0	0	0	0	0	0	0	0	0	0.58	0	0	0	0	0.31
Friuli	0	0	0	0	0	0	1	0	0.41	0	0	0	0	0	0.36	0	0	0	0	0	0
Lazio	0	0	0	0	0	0	0	1	0	0	0	0	0	0	0	0	0	0	0	0	0
Liguria	0	0	0	0	0	0	0.41	0	1	0	0	0	0	0	0.08	0	0	0	0	0	0
Lombardia	0	0	0	0	0	0	0	0	0	1	0	0	0	0	0	0	0	0	0	0	0
Marche	0	0	0	0	0.85	0	0	0	0	0	1	0	0	0.68	0	0	0	0	0	0	0
Molise	0	0	0	0	0	0	0	0	0	0	0	1	0	0	0	0	0	0	0	0	0
Piemonte	0	0	0	0	0	0	0	0	0	0	0	0	1	0	0	0.05	0.16	0	0	0	0
Puglia	0	0	0	0	0.38	0	0	0	0	0	0.68	0	0	1	0	0	0	0	0	0	0
Sardegna	0	0	0	0.47	0	0	0.36	0	0.08	0	0	0	0	0	1	0	0	0	0	0	0
Sicilia	0	0	0	0	0	0.58	0	0	0	0	0	0	0.05	0	0	1	0	0	0	0	0.28
Toscana	0	0	0	0	0	0	0	0	0	0	0	0	0.16	0	0	0	1	0	0	0	0
Trento	0.17	0	0	0.82	0	0	0	0	0	0	0	0	0	0	0	0	0	1	0	0	0
Umbria	0	0	0	0	0	0	0	0	0	0	0	0	0	0	0	0	0	0	1	0	0
ValleAosta	0	0.15	0	0	0	0	0	0	0	0	0	0	0	0	0	0	0	0	0	1	0
Veneto	0	0	0	0	0	0.31	0	0	0	0	0	0	0	0	0	0.28	0	0	0	0	1

Table S11. Similarity Matrix of Total Hospitalised network in the first period.

	Abruzzo	Basilicata	Bolzano	Calabria	Campania	Emilia	Friuli	Lazio	Liguria	Lombardia	Marche	Molise	Piemonte	Puglia	Sardegna	Sicilia	Toscana	Trento	Umbria	ValleAosta	Veneto
Abruzzo	1	0	0	0	0	0	0	0	0	0	0	0	0	0	0	0.06	0	0	0	0	0
Basilicata	0	1	0	0	0	0	0	0	0	0	0	0	0	0	0	0	0	0	0	0	0
Bolzano	0	0	1	0	0	0	0.65	0	0	0	0	0	0	0	0	0	0	0.07	0	0	0
Calabria	0	0	0	1	0	0	0	0	0	0	0	0	0	0	0.61	0	0	0	0.72	0	0
Campania	0	0	0	0	1	0	0	0	0	0	0	0	0	0.61	0	0	0	0	0	0	0
Emilia	0	0	0	0	0	1	0	0	0	0	0	0	0.96	0	0	0	0	0	0	0	0
Friuli	0	0	0.65	0	0	0	1	0	0	0	0	0	0	0	0	0	0	0.13	0	0	0
Lazio	0	0	0	0	0	0	0	1	0	0	0	0	0	0	0	0	0	0	0	0	0.69
Liguria	0	0	0	0	0	0	0	0	1	0	0.23	0	0	0	0	0	0.93	0	0	0	0
Lombardia	0	0	0	0	0	0	0	0	0	1	0	0	0	0	0	0	0	0	0	0	0
Marche	0	0	0	0	0	0	0	0	0.23	0	1	0	0	0	0	0	0.21	0	0	0	0
Molise	0	0	0	0	0	0	0	0	0	0	0	1	0	0	0	0	0	0	0	0	0
Piemonte	0	0	0	0	0	0.96	0	0	0	0	0	0	1	0	0	0	0	0	0	0	0
Puglia	0	0	0	0	0.61	0	0	0	0	0	0	0	0	1	0	0	0	0	0	0	0
Sardegna	0	0	0	0.61	0	0	0	0	0	0	0	0	0	0	1	0	0	0	0.97	0	0
Sicilia	0.06	0	0	0	0	0	0	0	0	0	0	0	0	0	0	1	0	0	0	0	0
Toscana	0	0	0	0	0	0	0	0	0.93	0	0.21	0	0	0	0	0	1	0	0	0	0
Trento	0	0	0.07	0	0	0	0.13	0	0	0	0	0	0	0	0	0	0	1	0	0	0
Umbria	0	0	0	0.72	0	0	0	0	0	0	0	0	0	0	0.97	0	0	0	1	0.06	0
ValleAosta	0	0	0	0	0	0	0	0	0	0	0	0	0	0	0	0	0	0	0.06	1	0
Veneto	0	0	0	0	0	0	0	0.69	0	0	0	0	0	0	0	0	0	0	0	0	1

Table S12. Similarity Matrix of Total Hospitalised network in the second period.

	Abruzzo	Basilicata	Bolzano	Calabria	Campania	Emilia	Friuli	Lazio	Liguria	Lombardia	Marche	Molise	Piemonte	Puglia	Sardegna	Sicilia	Toscana	Trento	Umbria	ValleAosta	Veneto
Abruzzo	1	0	0	0	0	0	0	0	0	0	0	0	0	0	0.13	0	0	0	0	0	0
Basilicata	0	1	0	0	0	0	0	0	0	0	0	0	0	0	0	0	0	0	0	0.22	0
Bolzano	0	0	1	0.57	0	0	0	0	0	0	0	0	0	0	0	0	0	0.69	0.63	0	0
Calabria	0	0	0.57	1	0	0	0	0	0	0	0	0	0	0	0	0	0	0.24	0.17	0	0
Campania	0	0	0	0	1	0	0	0	0	0	0	0	0	0	0	0	0	0	0	0	0.07
Emilia	0	0	0	0	0	1	0	0	0	0	0	0	0	0	0	0	0	0	0	0	0.18
Friuli	0	0	0	0	0	0	1	0	0	0	0.29	0	0	0	0.97	0	0	0	0	0	0
Lazio	0	0	0	0	0	0	0	1	0	0	0	0	0	0	0	0	0	0	0	0	0
Liguria	0	0	0	0	0	0	0	0	1	0	0	0	0	0	0	0	0	0	0	0	0
Lombardia	0	0	0	0	0	0	0	0	0	1	0	0	0	0	0	0	0	0	0	0	0
Marche	0	0	0	0	0	0	0.29	0	0	0	1	0	0	0	0.27	0	0	0	0	0	0
Molise	0	0	0	0	0	0	0	0	0	0	0	1	0	0	0	0	0	0	0	0	0
Piemonte	0	0	0	0	0	0	0	0	0	0	0	0	1	0	0	0	0	0	0	0	0
Puglia	0	0	0	0	0	0	0	0	0	0	0	0	0	1	0	0.66	0.83	0	0	0	0
Sardegna	0.13	0	0	0	0	0	0.97	0	0	0	0.27	0	0	0	1	0	0	0	0	0	0
Sicilia	0	0	0	0	0	0	0	0	0	0	0	0	0	0.66	0	1	0.74	0	0	0	0
Toscana	0	0	0	0	0	0	0	0	0	0	0	0	0	0.83	0	0.74	1	0	0	0	0
Trento	0	0	0.69	0.24	0	0	0	0	0	0	0	0	0	0	0	0	0	1	0.31	0	0
Umbria	0	0	0.63	0.17	0	0	0	0	0	0	0	0	0	0	0	0	0	0.31	1	0	0
ValleAosta	0	0.22	0	0	0	0	0	0	0	0	0	0	0	0	0	0	0	0	0	1	0
Veneto	0	0	0	0	0.07	0.18	0	0	0	0	0	0	0	0	0	0	0	0	0	0	1

Table S13. Similarity Matrix of Total Hospitalised network in the third period.

	Abruzzo	Basilicata	Bolzano	Calabria	Campania	Emilia	Friuli	Lazio	Liguria	Lombardia	Marche	Molise	Piemonte	Puglia	Sardegna	Sicilia	Toscana	Trento	Umbria	ValleAosta	Veneto
Abruzzo	1	0	0	0	0	0	0.45	0	0	0	0	0	0	0	0	0	0	0	0	0	0
Basilicata	0	1	0	0	0	0	0	0	0	0	0	0	0	0	0	0	0	0	0	0	0
Bolzano	0	0	1	0	0	0	0	0	0	0	0	0	0	0	0	0	0	0	0	0	0
Calabria	0	0	0	1	0	0	0	0	0	0	0	0	0	0	0.85	0	0	0	0.48	0	0
Campania	0	0	0	0	1	0	0	0	0	0	0	0	0	0	0	0	0	0	0	0	0
Emilia	0	0	0	0	0	1	0	0.37	0	0	0	0	0.66	0	0	0	0	0	0	0	0
Friuli	0.45	0	0	0	0	0	1	0	0	0	0	0	0	0	0	0	0	0	0	0	0
Lazio	0	0	0	0	0	0.37	0	1	0	0	0	0	0.48	0	0	0	0	0	0	0	0
Liguria	0	0	0	0	0	0	0	0	1	0	0.86	0	0	0	0	0	0	0	0	0	0
Lombardia	0	0	0	0	0	0	0	0	0	1	0	0	0	0	0	0	0	0	0	0	0
Marche	0	0	0	0	0	0	0	0	0.86	0	1	0	0	0	0	0	0	0	0	0	0
Molise	0	0	0	0	0	0	0	0	0	0	0	1	0	0	0	0	0	0	0	0	0
Piemonte	0	0	0	0	0	0.66	0	0.48	0	0	0	0	1	0	0	0	0	0	0	0	0
Puglia	0	0	0	0	0	0	0	0	0	0	0	0	0	1	0	0	0	0	0	0	0
Sardegna	0	0	0	0.85	0	0	0	0	0	0	0	0	0	0	1	0	0	0	0.88	0	0
Sicilia	0	0	0	0	0	0	0	0	0	0	0	0	0	0	0	1	0.4	0	0	0	0
Toscana	0	0	0	0	0	0	0	0	0	0	0	0	0	0	0	0.4	1	0	0	0	0
Trento	0	0	0	0	0	0	0	0	0	0	0	0	0	0	0	0	0	1	0	0	0
Umbria	0	0	0	0.48	0	0	0	0	0	0	0	0	0	0	0.88	0	0	0	1	0	0
ValleAosta	0	0	0	0	0	0	0	0	0	0	0	0	0	0	0	0	0	0	0	1	0
Veneto	0	0	0	0	0	0	0	0	0	0	0	0	0	0	0	0	0	0	0	0	1

Table S14. Similarity Matrix of Total Hospitalised network in the fourth period.

	Abruzzo	Basilicata	Bolzano	Calabria	Campania	Emilia	Friuli	Lazio	Liguria	Lombardia	Marche	Molise	Piemonte	Puglia	Sardegna	Sicilia	Toscana	Trento	Umbria	ValleAosta	Veneto
Abruzzo	1	0	0	0	0	0	0	0	0.09	0	0.7	0	0	0	0	0	0	0	0	0	0
Basilicata	0	1	0	0	0	0	0	0	0	0	0	0	0	0	0	0	0	0	0	0	0
Bolzano	0	0	1	0	0	0	0.45	0	0	0	0	0	0	0	0	0	0	0.41	0	0	0
Calabria	0	0	0	1	0	0	0	0	0	0	0	0	0	0	0.12	0	0	0	0	0	0
Campania	0	0	0	0	1	0.54	0	0	0	0	0	0	0	0	0	0	0	0	0	0	0
Emilia	0	0	0	0	0.54	1	0	0	0	0	0	0	0	0	0	0	0	0	0	0	0
Friuli	0	0	0.45	0	0	0	1	0	0	0	0	0	0	0	0	0	0	0	0	0	0
Lazio	0	0	0	0	0	0	0	1	0	0.27	0	0	0	0	0	0.69	0	0	0	0	0
Liguria	0.09	0	0	0	0	0	0	0	1	0	0	0	0	0	0	0	0	0	0	0	0
Lombardia	0	0	0	0	0	0	0	0.27	0	1	0	0	0	0	0	0.55	0	0	0	0	0
Marche	0.7	0	0	0	0	0	0	0	0	0	1	0	0	0	0	0	0	0	0	0	0
Molise	0	0	0	0	0	0	0	0	0	0	0	1	0	0	0	0	0	0	0	0	0
Piemonte	0	0	0	0	0	0	0	0	0	0	0	0	1	0.91	0	0	0	0	0	0	0
Puglia	0	0	0	0	0	0	0	0	0	0	0	0	0.91	1	0	0	0	0	0	0	0
Sardegna	0	0	0	0.12	0	0	0	0	0	0	0	0	0	0	1	0	0	0	0	0	0.1
Sicilia	0	0	0	0	0	0	0	0.69	0	0.55	0	0	0	0	0	1	0	0	0	0	0
Toscana	0	0	0	0	0	0	0	0	0	0	0	0	0	0	0	0	1	0	0	0	0
Trento	0	0	0.41	0	0	0	0	0	0	0	0	0	0	0	0	0	0	1	0	0	0
Umbria	0	0	0	0	0	0	0	0	0	0	0	0	0	0	0	0	0	0	1	0	0
ValleAosta	0	0	0	0	0	0	0	0	0	0	0	0	0	0	0	0	0	0	0	1	0
Veneto	0	0	0	0	0	0	0	0	0	0	0	0	0	0	0.1	0	0	0	0	0	1

Table S15. Similarity Matrix of Total Hospitalised network in the fifth period.

	Abruzzo	Basilicata	Bolzano	Calabria	Campania	Emilia	Friuli	Lazio	Liguria	Lombardia	Marche	Molise	Piemonte	Puglia	Sardegna	Sicilia	Toscana	Trento	Umbria	ValleAosta	Veneto
Abruzzo	1	0	0.2	0	0	0	0.43	0	0	0	0	0	0	0	0.08	0	0	0.17	0	0	0
Basilicata	0	1	0	0	0	0	0	0	0	0	0	0	0	0	0	0	0	0	0	0	0
Bolzano	0.2	0	1	0	0	0	0	0	0	0	0	0	0	0	0	0	0	0.23	0	0	0
Calabria	0	0	0	1	0	0	0.25	0	0	0	0	0	0	0	0	0	0	0	0	0	0
Campania	0	0	0	0	1	0	0	0	0	0	0	0	0	0	0	0	0.65	0	0	0	0.18
Emilia	0	0	0	0	0	1	0	0	0	0	0	0	0.69	0	0	0	0	0	0	0	0
Friuli	0.43	0	0	0.25	0	0	1	0	0	0	0	0	0	0	0.72	0	0	0	0	0	0
Lazio	0	0	0	0	0	0	0	1	0	0.09	0	0	0.13	0	0	0	0	0	0	0	0
Liguria	0	0	0	0	0	0	0	0	1	0	0	0	0	0	0	0	0	0	0	0	0
Lombardia	0	0	0	0	0	0	0	0.09	0	1	0	0	0	0	0	0	0	0	0	0	0
Marche	0	0	0	0	0	0	0	0	0	0	1	0	0	0	0	0	0	0	0	0	0
Molise	0	0	0	0	0	0	0	0	0	0	0	1	0	0	0	0	0	0	0	0	0
Piemonte	0	0	0	0	0	0.69	0	0.13	0	0	0	0	1	0	0	0	0	0	0	0	0
Puglia	0	0	0	0	0	0	0	0	0	0	0	0	0	1	0	0	0	0	0	0	0
Sardegna	0.08	0	0	0	0	0	0.72	0	0	0	0	0	0	0	1	0	0	0	0	0	0
Sicilia	0	0	0	0	0	0	0	0	0	0	0	0	0	0	0	1	0	0	0	0	0
Toscana	0	0	0	0	0.65	0	0	0	0	0	0	0	0	0	0	0	1	0	0	0	0.23
Trento	0.17	0	0.23	0	0	0	0	0	0	0	0	0	0	0	0	0	0	1	0.73	0	0
Umbria	0	0	0	0	0	0	0	0	0	0	0	0	0	0	0	0	0	0.73	1	0	0
ValleAosta	0	0	0	0	0	0	0	0	0	0	0	0	0	0	0	0	0	0	0	1	0
Veneto	0	0	0	0	0.18	0	0	0	0	0	0	0	0	0	0	0	0.23	0	0	0	1

Table S16. Similarity Matrix of Home Isolation network in the first period.

	Abruzzo	Basilicata	Bolzano	Calabria	Campania	Emilia	Friuli	Lazio	Liguria	Lombardia	Marche	Molise	Piemonte	Puglia	Sardegna	Sicilia	Toscana	Trento	Umbria	ValleAosta	Veneto
Abruzzo	1	0	0	0	0	0	0	0	0	0	0	0	0	0	0	0	0	0.22	0	0	0
Basilicata	0	1	0	0	0	0	0	0	0	0	0	0.06	0	0	0	0	0	0	0	0.17	0
Bolzano	0	0	1	0.07	0	0	0	0	0	0	0	0	0	0	0.11	0	0	0	0	0	0
Calabria	0	0	0.07	1	0	0	0	0	0	0	0	0	0	0	0.43	0	0	0	0	0	0
Campania	0	0	0	0	1	0	0	0	0.12	0	0	0	0	0.77	0	0.09	0	0	0	0	0
Emilia	0	0	0	0	0	1	0	0	0	0	0	0	0.22	0	0	0	0	0	0	0	0.11
Friuli	0	0	0	0	0	0	1	0	0	0	0	0	0	0	0	0	0	0.39	0	0	0
Lazio	0	0	0	0	0	0	0	1	0	0	0.25	0	0	0	0	0	0	0	0	0	0
Liguria	0	0	0	0	0.12	0	0	0	1	0	0.09	0	0	0.11	0	0	0	0	0	0	0
Lombardia	0	0	0	0	0	0	0	0	0	1	0	0	0	0	0	0	0	0	0	0	0
Marche	0	0	0	0	0	0	0	0.25	0.09	0	1	0	0	0	0	0	0	0	0	0	0
Molise	0	0.06	0	0	0	0	0	0	0	0	0	1	0	0	0	0	0	0	0	0.44	0
Piemonte	0	0	0	0	0	0.22	0	0	0	0	0	0	1	0	0	0	0	0	0	0	0
Puglia	0	0	0	0	0.77	0	0	0	0.11	0	0	0	0	1	0	0	0	0	0	0	0
Sardegna	0	0	0.11	0.43	0	0	0	0	0	0	0	0	0	0	1	0	0	0	0	0	0
Sicilia	0	0	0	0	0.09	0	0	0	0	0	0	0	0	0	0	1	0	0	0	0	0
Toscana	0	0	0	0	0	0	0	0	0	0	0	0	0	0	0	0	1	0	0	0	0
Trento	0.22	0	0	0	0	0	0.39	0	0	0	0	0	0	0	0	0	0	1	0	0	0
Umbria	0	0	0	0	0	0	0	0	0	0	0	0	0	0	0	0	0	0	1	0.05	0
ValleAosta	0	0.17	0	0	0	0	0	0	0	0	0	0.44	0	0	0	0	0	0	0.05	1	0
Veneto	0	0	0	0	0	0.11	0	0	0	0	0	0	0	0	0	0	0	0	0	0	1

Table S17. Similarity Matrix of Home Isolation network in the second period.

	Abruzzo	Basilicata	Bolzano	Calabria	Campania	Emilia	Friuli	Lazio	Liguria	Lombardia	Marche	Molise	Piemonte	Puglia	Sardegna	Sicilia	Toscana	Trento	Umbria	ValleAosta	Veneto
Abruzzo	1	0	0	0	0	0	0.09	0	0	0	0.38	0	0	0	0.95	0	0	0	0	0	0
Basilicata	0	1	0	0	0	0	0	0	0	0	0	0	0	0	0	0	0	0	0	0	0
Bolzano	0	0	1	0	0	0	0	0	0.44	0	0	0	0	0	0	0	0	0	0	0	0
Calabria	0	0	0	1	0	0	0	0	0	0	0	0	0	0	0	0	0	0	0.55	0	0
Campania	0	0	0	0	1	0	0	0	0	0	0	0	0	0	0	0	0	0	0	0	0
Emilia	0	0	0	0	0	1	0	0	0	0	0	0	0.63	0	0	0	0	0	0	0	0
Friuli	0.09	0	0	0	0	0	1	0	0.54	0	0.5	0	0	0	0	0	0	0	0	0	0
Lazio	0	0	0	0	0	0	0	1	0	0	0	0	0	0	0	0	0	0	0	0	0.62
Liguria	0	0	0.44	0	0	0	0.54	0	1	0	0.18	0	0	0	0	0	0	0	0	0	0
Lombardia	0	0	0	0	0	0	0	0	0	1	0	0	0	0	0	0	0	0	0	0	0
Marche	0.38	0	0	0	0	0	0.5	0	0.18	0	1	0	0	0	0.28	0	0	0	0	0	0
Molise	0	0	0	0	0	0	0	0	0	0	0	1	0	0	0	0	0	0.7	0	0	0
Piemonte	0	0	0	0	0	0.63	0	0	0	0	0	0	1	0	0	0	0	0	0	0	0
Puglia	0	0	0	0	0	0	0	0	0	0	0	0	0	1	0	0.29	0.62	0	0	0	0
Sardegna	0.95	0	0	0	0	0	0	0	0	0	0.28	0	0	0	1	0	0	0	0	0	0
Sicilia	0	0	0	0	0	0	0	0	0	0	0	0	0	0.29	0	1	0.67	0	0	0	0
Toscana	0	0	0	0	0	0	0	0	0	0	0	0	0	0.62	0	0.67	1	0	0	0	0
Trento	0	0	0	0	0	0	0	0	0	0	0	0.7	0	0	0	0	0	1	0	0	0
Umbria	0	0	0	0.55	0	0	0	0	0	0	0	0	0	0	0	0	0	0	1	0	0
ValleAosta	0	0	0	0	0	0	0	0	0	0	0	0	0	0	0	0	0	0	0	1	0
Veneto	0	0	0	0	0	0	0	0.62	0	0	0	0	0	0	0	0	0	0	0	0	1

Table S18. Similarity Matrix of Home Isolation network in the third period.

	Abruzzo	Basilicata	Bolzano	Calabria	Campania	Emilia	Friuli	Lazio	Liguria	Lombardia	Marche	Molise	Piemonte	Puglia	Sardegna	Sicilia	Toscana	Trento	Umbria	ValleAosta	Veneto
Abruzzo	1	0	0	0.45	0	0	0.23	0	0	0	0	0	0	0	0	0	0	0	0	0	0
Basilicata	0	1	0	0	0	0	0	0	0.15	0	0	0	0	0	0	0	0	0	0	0	0
Bolzano	0	0	1	0	0	0	0	0	0	0	0	0	0	0	0	0	0	0	0	0	0
Calabria	0.45	0	0	1	0	0	0.14	0	0	0	0	0	0	0	0	0	0	0	0	0	0
Campania	0	0	0	0	1	0	0	0	0	0	0	0	0	0	0	0	0	0	0	0	0
Emilia	0	0	0	0	0	1	0	0.26	0	0	0	0	0	0	0	0	0	0	0	0	0
Friuli	0.23	0	0	0.14	0	0	1	0	0	0	0	0	0	0	0	0	0	0	0	0	0
Lazio	0	0	0	0	0	0.26	0	1	0	0	0	0	0	0.57	0	0	0	0	0	0	0
Liguria	0	0.15	0	0	0	0	0	0	1	0	0	0	0	0	0	0	0	0	0.19	0	0
Lombardia	0	0	0	0	0	0	0	0	0	1	0	0	0	0	0	0	0	0	0	0	0
Marche	0	0	0	0	0	0	0	0	0	0	1	0	0	0	0	0	0	0	0	0	0
Molise	0	0	0	0	0	0	0	0	0	0	0	1	0	0	0	0	0	0	0	0	0
Piemonte	0	0	0	0	0	0	0	0	0	0	0	0	1	0	0	0	0.74	0	0	0	0
Puglia	0	0	0	0	0	0	0	0.57	0	0	0	0	0	1	0	0	0	0	0	0	0
Sardegna	0	0	0	0	0	0	0	0	0	0	0	0	0	0	1	0	0.75	0	0	0	0
Sicilia	0	0	0	0	0	0	0	0	0	0	0	0	0	0	0	1	0	0	0	0	0
Toscana	0	0	0	0	0	0	0	0	0	0	0	0	0.74	0	0.75	0	1	0	0	0	0
Trento	0	0	0	0	0	0	0	0	0	0	0	0	0	0	0	0	0	1	0	0	0
Umbria	0	0	0	0	0	0	0	0	0.19	0	0	0	0	0	0	0	0	0	1	0	0
ValleAosta	0	0	0	0	0	0	0	0	0	0	0	0	0	0	0	0	0	0	0	1	0
Veneto	0	0	0	0	0	0	0	0	0	0	0	0	0	0	0	0	0	0	0	0	1

Table S19. Similarity Matrix of Home Isolation network in the fourth period.

	Abruzzo	Basilicata	Bolzano	Calabria	Campania	Emilia	Friuli	Lazio	Liguria	Lombardia	Marche	Molise	Piemonte	Puglia	Sardegna	Sicilia	Toscana	Trento	Umbria	ValleAosta	Veneto
Abruzzo	1	0	0	0	0	0	0	0	0	0	0	0	0	0	0	0	0	0	0	0	0
Basilicata	0	1	0	0	0	0	0.07	0	0.07	0	0	0	0	0	0	0	0	0	0	0	0
Bolzano	0	0	1	0	0	0	0	0	0	0	0	0	0	0	0	0	0	0	0	0	0
Calabria	0	0	0	1	0	0	0	0	0	0	0	0	0	0.58	0	0	0	0	0	0	0
Campania	0	0	0	0	1	0	0	0.45	0	0	0	0	0	0	0	0.39	0	0	0	0	0
Emilia	0	0	0	0	0	1	0	0.54	0	0	0	0	0	0	0	0	0	0	0	0	0.39
Friuli	0	0.07	0	0	0	0	1	0	0.94	0	0	0	0	0	0	0	0	0	0	0	0
Lazio	0	0	0	0	0.45	0.54	0	1	0	0	0	0	0	0	0	0	0	0	0	0	0.65
Liguria	0	0.07	0	0	0	0	0.94	0	1	0	0	0	0	0	0	0	0	0	0	0	0
Lombardia	0	0	0	0	0	0	0	0	0	1	0	0	0	0	0	0.13	0	0	0	0	0
Marche	0	0	0	0	0	0	0	0	0	0	1	0	0	0	0	0	0	0	0	0	0
Molise	0	0	0	0	0	0	0	0	0	0	0	1	0	0	0	0	0	0	0	0	0
Piemonte	0	0	0	0	0	0	0	0	0	0	0	0	1	0	0	0	0	0	0	0	0
Puglia	0	0	0	0.58	0	0	0	0	0	0	0	0	0	1	0	0	0	0	0	0	0
Sardegna	0	0	0	0	0	0	0	0	0	0	0	0	0	0	1	0	0.6	0	0	0	0
Sicilia	0	0	0	0	0.39	0	0	0	0	0.13	0	0	0	0	0	1	0	0	0	0	0
Toscana	0	0	0	0	0	0	0	0	0	0	0	0	0	0	0.6	0	1	0	0	0	0
Trento	0	0	0	0	0	0	0	0	0	0	0	0	0	0	0	0	0	1	0	0	0
Umbria	0	0	0	0	0	0	0	0	0	0	0	0	0	0	0	0	0	0	1	0	0
ValleAosta	0	0	0	0	0	0	0	0	0	0	0	0	0	0	0	0	0	0	0	1	0
Veneto	0	0	0	0	0	0.39	0	0.65	0	0	0	0	0	0	0	0	0	0	0	0	1

Table S20. Similarity Matrix of Home Isolation network in the fifth period.

	Abruzzo	Basilicata	Bolzano	Calabria	Campania	Emilia	Friuli	Lazio	Liguria	Lombardia	Marche	Molise	Piemonte	Puglia	Sardegna	Sicilia	Toscana	Trento	Umbria	ValleAosta	Veneto
Abruzzo	1	0	0	0	0	0	0	0	0	0	0	0	0	0	0.51	0	0	0.87	0	0	0
Basilicata	0	1	0	0	0	0	0	0	0	0	0	0.42	0	0	0	0	0	0	0	0	0
Bolzano	0	0	1	0	0	0	0	0	0	0	0	0	0	0	0	0	0	0	0	0	0
Calabria	0	0	0	1	0	0	0	0	0	0	0	0	0	0	0.11	0	0	0.57	0	0	0
Campania	0	0	0	0	1	0.09	0	0	0	0	0	0	0.19	0	0	0.96	0	0	0	0	0.89
Emilia	0	0	0	0	0.09	1	0	0	0	0	0	0	0.28	0	0	0	0	0	0	0	0.12
Friuli	0	0	0	0	0	0	1	0	0	0	0	0	0	0	0	0	0	0.25	0.62	0	0
Lazio	0	0	0	0	0	0	0	1	0	0.39	0	0	0	0	0	0	0	0	0	0	0
Liguria	0	0	0	0	0	0	0	0	1	0	0.32	0	0	0	0	0	0	0	0	0	0
Lombardia	0	0	0	0	0	0	0	0.39	0	1	0	0	0	0	0	0	0	0	0	0	0
Marche	0	0	0	0	0	0	0	0	0.32	0	1	0	0	0	0	0	0	0	0	0	0
Molise	0	0.42	0	0	0	0	0	0	0	0	0	1	0	0	0	0	0	0	0	0	0
Piemonte	0	0	0	0	0.19	0.28	0	0	0	0	0	0	1	0	0	0.72	0.27	0	0	0	0.67
Puglia	0	0	0	0	0	0	0	0	0	0	0	0	0	1	0	0	0	0	0	0	0
Sardegna	0.51	0	0	0.11	0	0	0	0	0	0	0	0	0	0	1	0	0	0.29	0	0	0
Sicilia	0	0	0	0	0.96	0	0	0	0	0	0	0	0.72	0	0	1	0	0	0	0	0.4
Toscana	0	0	0	0	0	0	0	0	0	0	0	0	0.27	0	0	0	1	0	0	0	0
Trento	0.87	0	0	0.57	0	0	0.25	0	0	0	0	0	0	0	0.29	0	0	1	0.9	0	0
Umbria	0	0	0	0	0	0	0.62	0	0	0	0	0	0	0	0	0	0	0.9	1	0	0
ValleAosta	0	0	0	0	0	0	0	0	0	0	0	0	0	0	0	0	0	0	0	1	0
Veneto	0	0	0	0	0.89	0.12	0	0	0	0	0	0	0.67	0	0	0.4	0	0	0	0	1

Table S21. Similarity Matrix of Total Currently Positive network in the first period.

	Abruzzo	Basilicata	Bolzano	Calabria	Campania	Emilia	Friuli	Lazio	Liguria	Lombardia	Marche	Molise	Piemonte	Puglia	Sardegna	Sicilia	Toscana	Trento	Umbria	ValleAosta	Veneto
Abruzzo	1	0	0	0	0	0	0	0	0	0	0	0	0	0	0	0	0	0.11	0	0	0
Basilicata	0	1	0	0	0	0	0	0	0	0	0	0.96	0	0	0	0	0	0	0	0	0
Bolzano	0	0	1	0	0	0	0.09	0	0	0	0	0	0	0	0.06	0	0	0	0	0	0
Calabria	0	0	0	1	0	0	0	0	0	0	0	0	0	0	0.56	0	0	0	0	0	0
Campania	0	0	0	0	1	0	0	0	0	0	0	0	0	0.85	0	0.06	0	0	0	0	0
Emilia	0	0	0	0	0	1	0	0	0	0	0	0	0.14	0	0	0	0	0	0	0	0
Friuli	0	0	0.09	0	0	0	1	0	0	0	0	0	0	0	0	0	0	0.24	0	0	0
Lazio	0	0	0	0	0	0	0	1	0	0	0	0	0	0	0	0	0.06	0	0	0	0
Liguria	0	0	0	0	0	0	0	0	1	0	0.63	0	0	0	0	0	0	0	0	0	0
Lombardia	0	0	0	0	0	0	0	0	0	1	0	0	0	0	0	0	0	0	0	0	0
Marche	0	0	0	0	0	0	0	0	0.63	0	1	0	0	0	0	0	0	0	0	0	0
Molise	0	0.96	0	0	0	0	0	0	0	0	0	1	0	0	0	0	0	0	0	0.17	0
Piemonte	0	0	0	0	0	0.14	0	0	0	0	0	0	1	0	0	0	0	0	0	0	0
Puglia	0	0	0	0	0.85	0	0	0	0	0	0	0	0	1	0	0	0	0	0	0	0
Sardegna	0	0	0.06	0.56	0	0	0	0	0	0	0	0	0	0	1	0	0	0	0	0	0
Sicilia	0	0	0	0	0.06	0	0	0	0	0	0	0	0	0	0	1	0	0	0	0	0
Toscana	0	0	0	0	0	0	0	0.06	0	0	0	0	0	0	0	0	1	0	0	0	0
Trento	0.11	0	0	0	0	0	0.24	0	0	0	0	0	0	0	0	0	0	1	0	0	0
Umbria	0	0	0	0	0	0	0	0	0	0	0	0	0	0	0	0	0	0	1	0.1	0
ValleAosta	0	0	0	0	0	0	0	0	0	0	0	0.17	0	0	0	0	0	0	0.1	1	0
Veneto	0	0	0	0	0	0	0	0	0	0	0	0	0	0	0	0	0	0	0	0	1

Table S22. Similarity Matrix of Total Currently Positive network in the second period.

	Abruzzo	Basilicata	Bolzano	Calabria	Campania	Emilia	Friuli	Lazio	Liguria	Lombardia	Marche	Molise	Piemonte	Puglia	Sardegna	Sicilia	Toscana	Trento	Umbria	ValleAosta	Veneto
Abruzzo	1	0	0	0	0	0	0.08	0	0.09	0	0.33	0	0	0	1	0	0	0	0	0	0
Basilicata	0	1	0	0	0	0	0	0	0	0	0	0	0	0	0	0	0	0	0	0	0
Bolzano	0	0	1	0	0	0	0	0	0.15	0	0	0	0	0	0	0	0	0	0	0	0
Calabria	0	0	0	1	0	0	0	0	0	0	0	0	0	0	0	0	0	0	0.59	0	0
Campania	0	0	0	0	1	0	0	0	0	0	0	0	0	0	0	0	0	0	0	0	0
Emilia	0	0	0	0	0	1	0	0	0	0	0	0	0.92	0	0	0	0	0	0	0	0
Friuli	0.08	0	0	0	0	0	1	0	0.97	0	0.51	0	0	0	0	0	0	0	0	0	0
Lazio	0	0	0	0	0	0	0	1	0	0	0	0	0	0	0	0	0	0	0	0	0.5
Liguria	0.09	0	0.15	0	0	0	0.97	0	1	0	0.42	0	0	0	0.08	0	0	0	0	0	0
Lombardia	0	0	0	0	0	0	0	0	0	1	0	0	0	0	0	0	0	0	0	0	0
Marche	0.33	0	0	0	0	0	0.51	0	0.42	0	1	0	0	0	0.27	0	0	0	0	0	0
Molise	0	0	0	0	0	0	0	0	0	0	0	1	0	0	0	0	0	0	0	0	0
Piemonte	0	0	0	0	0	0.92	0	0	0	0	0	0	1	0	0	0	0	0	0	0	0
Puglia	0	0	0	0	0	0	0	0	0	0	0	0	0	1	0	0.28	0.66	0	0	0	0
Sardegna	1	0	0	0	0	0	0	0	0.08	0	0.27	0	0	0	1	0	0	0	0	0	0
Sicilia	0	0	0	0	0	0	0	0	0	0	0	0	0	0.28	0	1	0.7	0	0	0	0
Toscana	0	0	0	0	0	0	0	0	0	0	0	0	0	0.66	0	0.7	1	0	0	0	0
Trento	0	0	0	0	0	0	0	0	0	0	0	0	0	0	0	0	0	1	0	0	0
Umbria	0	0	0	0.59	0	0	0	0	0	0	0	0	0	0	0	0	0	0	1	0	0
ValleAosta	0	0	0	0	0	0	0	0	0	0	0	0	0	0	0	0	0	0	0	1	0
Veneto	0	0	0	0	0	0	0	0.5	0	0	0	0	0	0	0	0	0	0	0	0	1

Table S23. Similarity Matrix of Total Currently Positive network in the third period.

	Abruzzo	Basilicata	Bolzano	Calabria	Campania	Emilia	Friuli	Lazio	Liguria	Lombardia	Marche	Molise	Piemonte	Puglia	Sardegna	Sicilia	Toscana	Trento	Umbria	ValleAosta	Veneto
Abruzzo	1	0	0	0.14	0	0	0.24	0	0	0	0	0	0	0	0	0	0	0	0	0	0
Basilicata	0	1	0	0	0	0	0	0	0.12	0	0	0	0	0	0	0	0	0	0.08	0	0
Bolzano	0	0	1	0	0	0	0	0	0	0	0	0	0	0	0	0	0	0	0	0	0
Calabria	0.14	0	0	1	0	0	0.07	0	0	0	0	0	0	0	0	0	0	0	0	0	0
Campania	0	0	0	0	1	0	0	0	0	0	0	0	0	0	0	0	0	0	0	0	0
Emilia	0	0	0	0	0	1	0	0.31	0	0	0	0	0	0	0	0	0	0	0	0	0
Friuli	0.24	0	0	0.07	0	0	1	0	0	0	0	0	0	0	0	0	0	0	0	0	0
Lazio	0	0	0	0	0	0.31	0	1	0	0	0	0	0	0.18	0	0	0	0	0	0	0
Liguria	0	0.12	0	0	0	0	0	0	1	0	0	0	0	0	0	0	0	0	0	0	0
Lombardia	0	0	0	0	0	0	0	0	0	1	0	0	0	0	0	0	0	0	0	0	0
Marche	0	0	0	0	0	0	0	0	0	0	1	0	0	0	0	0	0	0	0	0	0
Molise	0	0	0	0	0	0	0	0	0	0	0	1	0	0	0	0	0	0	0	0	0
Piemonte	0	0	0	0	0	0	0	0	0	0	0	0	1	0	0.3	0	0.12	0	0	0	0
Puglia	0	0	0	0	0	0	0	0.18	0	0	0	0	0	1	0	0	0	0	0	0	0
Sardegna	0	0	0	0	0	0	0	0	0	0	0	0	0.3	0	1	0	0.32	0	0	0	0
Sicilia	0	0	0	0	0	0	0	0	0	0	0	0	0	0	0	1	0	0	0	0	0
Toscana	0	0	0	0	0	0	0	0	0	0	0	0	0.12	0	0.32	0	1	0	0	0	0
Trento	0	0	0	0	0	0	0	0	0	0	0	0	0	0	0	0	0	1	0	0	0
Umbria	0	0.08	0	0	0	0	0	0	0	0	0	0	0	0	0	0	0	0	1	0	0
ValleAosta	0	0	0	0	0	0	0	0	0	0	0	0	0	0	0	0	0	0	0	1	0
Veneto	0	0	0	0	0	0	0	0	0	0	0	0	0	0	0	0	0	0	0	0	1

Table S24. Similarity Matrix of Total Currently Positive network in the fourth period.

	Abruzzo	Basilicata	Bolzano	Calabria	Campania	Emilia	Friuli	Lazio	Liguria	Lombardia	Marche	Molise	Piemonte	Puglia	Sardegna	Sicilia	Toscana	Trento	Umbria	ValleAosta	Veneto
Abruzzo	1	0	0	0	0	0	0	0	0	0	0	0	0	0	0	0	0	0	0	0	0
Basilicata	0	1	0	0	0	0	0.09	0	0.11	0	0	0	0	0	0	0	0	0	0	0	0
Bolzano	0	0	1	0	0	0	0	0	0	0	0	0	0	0	0	0	0	0	0	0	0
Calabria	0	0	0	1	0	0	0	0	0	0	0	0	0	0.74	0	0	0	0	0	0	0
Campania	0	0	0	0	1	0	0	0.6	0	0	0	0	0	0	0	0.46	0	0	0	0	0
Emilia	0	0	0	0	0	1	0	0.45	0	0	0	0	0	0	0	0	0	0	0	0	0.53
Friuli	0	0.09	0	0	0	0	1	0	0.8	0	0	0	0	0	0	0	0	0	0	0	0
Lazio	0	0	0	0	0.6	0.45	0	1	0	0	0	0	0	0	0	0	0	0	0	0	0.9
Liguria	0	0.11	0	0	0	0	0.8	0	1	0	0	0	0	0	0	0	0	0	0	0	0
Lombardia	0	0	0	0	0	0	0	0	0	1	0	0	0	0	0	0.14	0	0	0	0	0
Marche	0	0	0	0	0	0	0	0	0	0	1	0	0	0	0	0	0	0	0	0	0
Molise	0	0	0	0	0	0	0	0	0	0	0	1	0	0	0	0	0	0	0	0	0
Piemonte	0	0	0	0	0	0	0	0	0	0	0	0	1	0	0	0	0	0	0	0	0
Puglia	0	0	0	0.74	0	0	0	0	0	0	0	0	0	1	0.05	0	0	0	0	0	0
Sardegna	0	0	0	0	0	0	0	0	0	0	0	0	0	0.05	1	0	0.38	0	0	0	0
Sicilia	0	0	0	0	0.46	0	0	0	0	0.14	0	0	0	0	0	1	0	0	0	0	0
Toscana	0	0	0	0	0	0	0	0	0	0	0	0	0	0	0.38	0	1	0	0	0	0
Trento	0	0	0	0	0	0	0	0	0	0	0	0	0	0	0	0	0	1	0	0	0
Umbria	0	0	0	0	0	0	0	0	0	0	0	0	0	0	0	0	0	0	1	0	0
ValleAosta	0	0	0	0	0	0	0	0	0	0	0	0	0	0	0	0	0	0	0	1	0
Veneto	0	0	0	0	0	0.53	0	0.9	0	0	0	0	0	0	0	0	0	0	0	0	1

Table S25. Similarity Matrix of Total Currently Positive network in the fifth period.

	Abruzzo	Basilicata	Bolzano	Calabria	Campania	Emilia	Friuli	Lazio	Liguria	Lombardia	Marche	Molise	Piemonte	Puglia	Sardegna	Sicilia	Toscana	Trento	Umbria	ValleAosta	Veneto
Abruzzo	1	0	0	0	0	0	0.05	0	0	0	0	0	0	0	0.48	0	0	0.86	0	0	0
Basilicata	0	1	0	0	0	0	0	0	0	0	0	0.42	0	0	0	0	0	0	0	0	0
Bolzano	0	0	1	0	0	0	0	0	0	0	0	0	0	0	0	0	0	0	0	0	0
Calabria	0	0	0	1	0	0	0	0	0	0	0	0	0	0	0.1	0	0	0.55	0	0	0
Campania	0	0	0	0	1	0.08	0	0	0	0	0	0	0.22	0	0	0.98	0	0	0	0	0.91
Emilia	0	0	0	0	0.08	1	0	0	0	0	0	0	0.28	0	0	0	0	0	0	0	0.1
Friuli	0.05	0	0	0	0	0	1	0	0	0	0	0	0	0	0	0	0	0.28	0.65	0	0
Lazio	0	0	0	0	0	0	0	1	0	0.4	0	0	0	0	0	0	0	0	0	0	0
Liguria	0	0	0	0	0	0	0	0	1	0	0.35	0	0	0	0	0	0	0	0	0	0
Lombardia	0	0	0	0	0	0	0	0.4	0	1	0	0	0	0	0	0	0	0	0	0	0
Marche	0	0	0	0	0	0	0	0	0.35	0	1	0	0	0	0	0	0	0	0	0	0
Molise	0	0.42	0	0	0	0	0	0	0	0	0	1	0	0	0	0	0	0	0	0	0
Piemonte	0	0	0	0	0.22	0.28	0	0	0	0	0	0	1	0	0	0.74	0.25	0	0	0	0.63
Puglia	0	0	0	0	0	0	0	0	0	0	0	0	0	1	0	0	0	0	0	0	0
Sardegna	0.48	0	0	0.1	0	0	0	0	0	0	0	0	0	0	1	0	0	0.28	0	0	0
Sicilia	0	0	0	0	0.98	0	0	0	0	0	0	0	0.74	0	0	1	0	0	0	0	0.46
Toscana	0	0	0	0	0	0	0	0	0	0	0	0	0.25	0	0	0	1	0	0	0	0
Trento	0.86	0	0	0.55	0	0	0.28	0	0	0	0	0	0	0	0.28	0	0	1	0.92	0	0
Umbria	0	0	0	0	0	0	0.65	0	0	0	0	0	0	0	0	0	0	0.92	1	0	0
ValleAosta	0	0	0	0	0	0	0	0	0	0	0	0	0	0	0	0	0	0	0	1	0
Veneto	0	0	0	0	0.91	0.1	0	0	0	0	0	0	0.63	0	0	0.46	0	0	0	0	1

Table S26. Similarity Matrix of New Currently Positive network in the first period.

	Abruzzo	Basilicata	Bolzano	Calabria	Campania	Emilia	Friuli	Lazio	Liguria	Lombardia	Marche	Molise	Piemonte	Puglia	Sardegna	Sicilia	Toscana	Trento	Umbria	ValleAosta	Veneto
Abruzzo	1	0	0	0	0	0	0.71	0	0	0	0	0	0	0	0	0.6	0	0.21	0	0	0
Basilicata	0	1	0	0	0	0	0	0	0	0	0	0.73	0	0	0	0	0	0	0	0	0
Bolzano	0	0	1	0	0	0	0	0	0	0	0	0	0	0	0.05	0	0	0	0	0	0
Calabria	0	0	0	1	0	0	0	0	0	0	0	0	0	0	0.7	0	0	0	0.85	0.81	0
Campania	0	0	0	0	1	0	0	0	0	0	0	0	0	0.65	0	0.1	0	0.28	0	0	0
Emilia	0	0	0	0	0	1	0	0	0	0	0	0	0.51	0	0	0	0	0	0	0	0
Friuli	0.71	0	0	0	0	0	1	0	0	0	0	0	0	0.11	0	0.85	0	0.46	0	0	0
Lazio	0	0	0	0	0	0	0	1	0.08	0	0.17	0	0	0	0	0	0.41	0	0	0	0
Liguria	0	0	0	0	0	0	0	0.08	1	0	0	0	0	0	0	0	0.58	0	0	0	0
Lombardia	0	0	0	0	0	0	0	0	0	1	0	0	0	0	0	0	0	0	0	0	0
Marche	0	0	0	0	0	0	0	0.17	0	0	1	0	0	0	0	0	0	0	0	0	0
Molise	0	0.73	0	0	0	0	0	0	0	0	0	1	0	0	0	0	0	0	0	0	0
Piemonte	0	0	0	0	0	0.51	0	0	0	0	0	0	1	0	0	0	0	0	0	0	0
Puglia	0	0	0	0	0.65	0	0.11	0	0	0	0	0	0	1	0	0.16	0	0.51	0	0	0
Sardegna	0	0	0.05	0.7	0	0	0	0	0	0	0	0	0	0	1	0	0	0	0.6	0.55	0
Sicilia	0.6	0	0	0	0.1	0	0.85	0	0	0	0	0	0	0.16	0	1	0	0.43	0	0	0
Toscana	0	0	0	0	0	0	0	0.41	0.58	0	0	0	0	0	0	0	1	0	0	0	0
Trento	0.21	0	0	0	0.28	0	0.46	0	0	0	0	0	0	0.51	0	0.43	0	1	0	0	0
Umbria	0	0	0	0.85	0	0	0	0	0	0	0	0	0	0	0.6	0	0	0	1	0.91	0
ValleAosta	0	0	0	0.81	0	0	0	0	0	0	0	0	0	0	0.55	0	0	0	0.91	1	0
Veneto	0	0	0	0	0	0	0	0	0	0	0	0	0	0	0	0	0	0	0	0	1

Table S27. Similarity Matrix of New Currently Positive network in the second period.

	Abruzzo	Basilicata	Bolzano	Calabria	Campania	Emilia	Friuli	Lazio	Liguria	Lombardia	Marche	Molise	Piemonte	Puglia	Sardegna	Sicilia	Toscana	Trento	Umbria	ValleAosta	Veneto
Abruzzo	1	0	0.07	0	0	0	0	0	0	0	0.31	0	0	0	0.31	0	0	0	0.12	0	0
Basilicata	0	1	0	0	0	0	0	0	0	0	0	0	0	0	0	0	0	0	0	0	0
Bolzano	0.07	0	1	0.4	0	0	0	0	0	0	0	0	0	0	0.09	0	0	0	0.78	0	0
Calabria	0	0	0.4	1	0	0	0	0	0	0	0	0	0	0	0	0	0	0	0.3	0	0
Campania	0	0	0	0	1	0.09	0	0.26	0	0	0	0	0.28	0	0	0	0	0	0	0	0
Emilia	0	0	0	0	0.09	1	0	0.38	0	0	0	0	0.55	0	0	0	0	0	0	0	0
Friuli	0	0	0	0	0	0	1	0	0.95	0	0	0	0	0	0	0	0	0	0	0	0
Lazio	0	0	0	0	0.26	0.38	0	1	0	0	0	0	0.92	0	0	0	0	0	0	0	0
Liguria	0	0	0	0	0	0	0.95	0	1	0	0	0	0	0	0	0	0	0	0	0	0
Lombardia	0	0	0	0	0	0	0	0	0	1	0	0	0	0	0	0	0	0	0	0	0
Marche	0.31	0	0	0	0	0	0	0	0	0	1	0	0	0	0	0	0	0	0	0	0
Molise	0	0	0	0	0	0	0	0	0	0	0	1	0	0	0	0	0	0	0	0.87	0
Piemonte	0	0	0	0	0.28	0.55	0	0.92	0	0	0	0	1	0	0	0	0	0	0	0	0
Puglia	0	0	0	0	0	0	0	0	0	0	0	0	0	1	0	0.69	0.31	0	0	0	0
Sardegna	0.31	0	0.09	0	0	0	0	0	0	0	0	0	0	0	1	0	0	0	0.18	0	0
Sicilia	0	0	0	0	0	0	0	0	0	0	0	0	0	0.69	0	1	0.77	0	0	0	0
Toscana	0	0	0	0	0	0	0	0	0	0	0	0	0	0.31	0	0.77	1	0	0	0	0
Trento	0	0	0	0	0	0	0	0	0	0	0	0	0	0	0	0	0	1	0	0	0
Umbria	0.12	0	0.78	0.3	0	0	0	0	0	0	0	0	0	0	0.18	0	0	0	1	0	0
ValleAosta	0	0	0	0	0	0	0	0	0	0	0	0.87	0	0	0	0	0	0	0	1	0
Veneto	0	0	0	0	0	0	0	0	0	0	0	0	0	0	0	0	0	0	0	0	1

Table S28. Similarity Matrix of New Currently Positive network in the third period.

	Abruzzo	Basilicata	Bolzano	Calabria	Campania	Emilia	Friuli	Lazio	Liguria	Lombardia	Marche	Molise	Piemonte	Puglia	Sardegna	Sicilia	Toscana	Trento	Umbria	ValleAosta	Veneto
Abruzzo	1	0	0	0.05	0	0	0	0	0	0	0	0	0	0	0	0	0	0	0	0	0
Basilicata	0	1	0	0	0	0	0	0	0	0	0	0	0	0	0	0	0	0	0	0	0
Bolzano	0	0	1	0	0	0	0	0	0	0	0	0	0	0	0.63	0	0	0.18	0.9	0	0
Calabria	0.05	0	0	1	0	0	0	0	0.7	0	0	0	0	0	0	0	0	0	0	0	0
Campania	0	0	0	0	1	0.09	0	0	0	0	0	0	0	0	0	0	0	0	0	0	0
Emilia	0	0	0	0	0.09	1	0	0	0	0	0	0	0	0	0	0	0	0	0	0	0
Friuli	0	0	0	0	0	0	1	0	0	0	0.12	0	0	0	0	0	0	0	0	0	0
Lazio	0	0	0	0	0	0	0	1	0	0	0	0	0	0	0	0	0	0	0	0	0
Liguria	0	0	0	0.7	0	0	0	0	1	0	0	0	0	0	0	0	0	0	0	0	0
Lombardia	0	0	0	0	0	0	0	0	0	1	0	0	0	0	0	0	0	0	0	0	0
Marche	0	0	0	0	0	0	0.12	0	0	0	1	0	0	0	0	0	0	0	0	0	0
Molise	0	0	0	0	0	0	0	0	0	0	0	1	0	0	0	0	0	0	0	0	0
Piemonte	0	0	0	0	0	0	0	0	0	0	0	0	1	0.5	0	0	0	0	0	0	0.89
Puglia	0	0	0	0	0	0	0	0	0	0	0	0	0.5	1	0	0	0	0	0	0	0.61
Sardegna	0	0	0.63	0	0	0	0	0	0	0	0	0	0	0	1	0	0	0.34	0.37	0	0
Sicilia	0	0	0	0	0	0	0	0	0	0	0	0	0	0	0	1	0.16	0	0	0	0
Toscana	0	0	0	0	0	0	0	0	0	0	0	0	0	0	0	0.16	1	0	0	0	0
Trento	0	0	0.18	0	0	0	0	0	0	0	0	0	0	0	0.34	0	0	1	0.06	0	0
Umbria	0	0	0.9	0	0	0	0	0	0	0	0	0	0	0	0.37	0	0	0.06	1	0	0
ValleAosta	0	0	0	0	0	0	0	0	0	0	0	0	0	0	0	0	0	0	0	1	0
Veneto	0	0	0	0	0	0	0	0	0	0	0	0	0.89	0.61	0	0	0	0	0	0	1

Table S29. Similarity Matrix of New Currently Positive network in the fourth period.

	Abruzzo	Basilicata	Bolzano	Calabria	Campania	Emilia	Friuli	Lazio	Liguria	Lombardia	Marche	Molise	Piemonte	Puglia	Sardegna	Sicilia	Toscana	Trento	Umbria	ValleAosta	Veneto
Abruzzo	1	0	0	0	0	0	0.11	0	0.29	0	0	0	0	0	0	0	0	0	0.49	0	0
Basilicata	0	1	0.82	0	0	0	0	0	0	0	0	0	0	0	0	0	0	0.23	0	0	0
Bolzano	0	0.82	1	0	0	0	0	0	0	0	0	0	0	0	0	0	0	0.24	0	0	0
Calabria	0	0	0	1	0	0	0	0	0	0	0	0	0	0	0.9	0	0	0	0	0	0
Campania	0	0	0	0	1	0.72	0	0.33	0	0	0	0	0	0	0	0	0.65	0	0	0	0.66
Emilia	0	0	0	0	0.72	1	0	0.34	0	0	0	0	0	0	0	0	0.53	0	0	0	0.52
Friuli	0.11	0	0	0	0	0	1	0	0	0	0	0	0	0	0	0	0	0	0.63	0	0
Lazio	0	0	0	0	0.33	0.34	0	1	0	0.08	0	0	0	0	0	0	0.77	0	0	0	0.93
Liguria	0.29	0	0	0	0	0	0	0	1	0	0.33	0	0	0	0	0	0	0	0.1	0	0
Lombardia	0	0	0	0	0	0	0	0.08	0	1	0	0	0	0	0	0	0.17	0	0	0	0.27
Marche	0	0	0	0	0	0	0	0	0.33	0	1	0	0	0	0	0	0	0	0	0	0
Molise	0	0	0	0	0	0	0	0	0	0	0	1	0	0	0	0	0	0	0	0.07	0
Piemonte	0	0	0	0	0	0	0	0	0	0	0	0	1	0.55	0.26	0	0	0	0	0	0
Puglia	0	0	0	0	0	0	0	0	0	0	0	0	0.55	1	0.3	0	0	0	0	0	0
Sardegna	0	0	0	0.9	0	0	0	0	0	0	0	0	0.26	0.3	1	0	0	0	0	0	0
Sicilia	0	0	0	0	0	0	0	0	0	0	0	0	0	0	0	1	0	0	0	0	0
Toscana	0	0	0	0	0.65	0.53	0	0.77	0	0.17	0	0	0	0	0	0	1	0	0	0	0.99
Trento	0	0.23	0.24	0	0	0	0	0	0	0	0	0	0	0	0	0	0	1	0	0	0
Umbria	0.49	0	0	0	0	0	0.63	0	0.1	0	0	0	0	0	0	0	0	0	1	0	0
ValleAosta	0	0	0	0	0	0	0	0	0	0	0	0.07	0	0	0	0	0	0	0	1	0
Veneto	0	0	0	0	0.66	0.52	0	0.93	0	0.27	0	0	0	0	0	0	0.99	0	0	0	1

Table S30. Similarity Matrix of New Currently Positive network in the fifth period.

	Abruzzo	Basilicata	Bolzano	Calabria	Campania	Emilia	Friuli	Lazio	Liguria	Lombardia	Marche	Molise	Piemonte	Puglia	Sardegna	Sicilia	Toscana	Trento	Umbria	ValleAosta	Veneto
Abruzzo	1	0	0	0	0	0	0	0	0	0	0	0	0	0	0.4	0	0	0	0.71	0	0
Basilicata	0	1	0	0	0	0	0	0	0	0	0	0.19	0	0	0	0	0	0	0	0	0
Bolzano	0	0	1	0	0	0	0	0	0	0	0	0	0	0	0	0	0	0	0	0	0
Calabria	0	0	0	1	0	0	0.17	0	0	0	0	0	0	0	0.96	0	0	0	0.16	0	0
Campania	0	0	0	0	1	0.36	0	0	0	0	0	0	0.54	0	0	0.09	0	0	0	0	0.28
Emilia	0	0	0	0	0.36	1	0	0	0	0	0	0	0.14	0.1	0	0.77	0.13	0	0	0	0.73
Friuli	0	0	0	0.17	0	0	1	0	0	0	0	0	0	0	0.61	0	0	0.26	0.32	0	0
Lazio	0	0	0	0	0	0	0	1	0	0.79	0	0	0	0	0	0	0	0	0	0	0
Liguria	0	0	0	0	0	0	0	0	1	0	0.09	0	0	0	0	0	0.1	0	0	0	0
Lombardia	0	0	0	0	0	0	0	0.79	0	1	0	0	0	0	0	0	0	0	0	0	0
Marche	0	0	0	0	0	0	0	0	0.09	0	1	0	0	0	0	0	0.19	0	0	0	0
Molise	0	0.19	0	0	0	0	0	0	0	0	0	1	0	0	0	0	0	0	0	0	0
Piemonte	0	0	0	0	0.54	0.14	0	0	0	0	0	0	1	0	0	0	0	0	0	0	0
Puglia	0	0	0	0	0	0.1	0	0	0	0	0	0	0	1	0	0.12	0.53	0	0	0	0.25
Sardegna	0.4	0	0	0.96	0	0	0.61	0	0	0	0	0	0	0	1	0	0	0.08	0.97	0	0
Sicilia	0	0	0	0	0.09	0.77	0	0	0	0	0	0	0	0.12	0	1	0.19	0	0	0	0.9
Toscana	0	0	0	0	0	0.13	0	0	0.1	0	0.19	0	0	0.53	0	0.19	1	0	0	0	0.1
Trento	0	0	0	0	0	0	0.26	0	0	0	0	0	0	0	0.08	0	0	1	0.09	0	0
Umbria	0.71	0	0	0.16	0	0	0.32	0	0	0	0	0	0	0	0.97	0	0	0.09	1	0	0
ValleAosta	0	0	0	0	0	0	0	0	0	0	0	0	0	0	0	0	0	0	0	1	0
Veneto	0	0	0	0	0.28	0.73	0	0	0	0	0	0	0	0.25	0	0.9	0.1	0	0	0	1

Table S31. Similarity Matrix of Discharged or Healed network in the first period.

	Abruzzo	Basilicata	Bolzano	Calabria	Campania	Emilia	Friuli	Lazio	Liguria	Lombardia	Marche	Molise	Piemonte	Puglia	Sardegna	Sicilia	Toscana	Trento	Umbria	ValleAosta	Veneto
Abruzzo	1	0	0.1	0	0	0	0	0	0	0	0	0	0	0.58	0.08	0.64	0	0	0.47	0.23	0
Basilicata	0	1	0	0	0	0	0	0	0	0	0	0.95	0	0	0	0	0	0	0	0	0
Bolzano	0.1	0	1	0	0.35	0	0.06	0	0	0	0	0	0	0.66	0	0.34	0	0	0.27	0	0
Calabria	0	0	0	1	0	0	0	0	0	0	0	0	0	0	0.33	0	0	0	0	0.07	0
Campania	0	0	0.35	0	1	0	0.53	0.15	0	0	0.64	0	0	0.11	0	0.1	0	0.14	0.16	0	0
Emilia	0	0	0	0	0	1	0	0	0	0	0	0	0.07	0	0	0	0	0	0	0	0.14
Friuli	0	0	0.06	0	0.53	0	1	0.46	0	0	0.62	0	0	0	0	0	0.16	0.36	0	0	0
Lazio	0	0	0	0	0.15	0	0.46	1	0	0	0.55	0	0	0	0	0	0.43	0.96	0	0	0
Liguria	0	0	0	0	0	0	0	0	1	0	0	0	0.14	0	0	0	0.37	0	0	0	0
Lombardia	0	0	0	0	0	0	0	0	0	1	0	0	0	0	0	0	0	0	0	0	0
Marche	0	0	0	0	0.64	0	0.62	0.55	0	0	1	0	0	0.06	0	0	0	0.6	0	0	0
Molise	0	0.95	0	0	0	0	0	0	0	0	0	1	0	0	0	0	0	0	0	0	0
Piemonte	0	0	0	0	0	0.07	0	0	0.14	0	0	0	1	0	0	0	0.07	0	0	0	0.49
Puglia	0.58	0	0.66	0	0.11	0	0	0	0	0	0.06	0	0	1	0	0.84	0	0	0.66	0.12	0
Sardegna	0.08	0	0	0.33	0	0	0	0	0	0	0	0	0	0	1	0	0	0	0	0.29	0
Sicilia	0.64	0	0.34	0	0.1	0	0	0	0	0	0	0	0	0.84	0	1	0	0	0.68	0.08	0
Toscana	0	0	0	0	0	0	0.16	0.43	0.37	0	0	0	0.07	0	0	0	1	0.19	0	0	0
Trento	0	0	0	0	0.14	0	0.36	0.96	0	0	0.6	0	0	0	0	0	0.19	1	0	0	0
Umbria	0.47	0	0.27	0	0.16	0	0	0	0	0	0	0	0	0.66	0	0.68	0	0	1	0	0
ValleAosta	0.23	0	0	0.07	0	0	0	0	0	0	0	0	0	0.12	0.29	0.08	0	0	0	1	0
Veneto	0	0	0	0	0	0.14	0	0	0	0	0	0	0.49	0	0	0	0	0	0	0	1

Table S32. Similarity Matrix of Discharged or Healed network in the second period.

	Abruzzo	Basilicata	Bolzano	Calabria	Campania	Emilia	Friuli	Lazio	Liguria	Lombardia	Marche	Molise	Piemonte	Puglia	Sardegna	Sicilia	Toscana	Trento	Umbria	ValleAosta	Veneto
Abruzzo	1	0	0.08	0	0	0	0	0	0	0	0	0	0	0	0	0	0	0	0.73	0	0
Basilicata	0	1	0	0	0	0	0	0	0	0	0	0.91	0	0	0	0	0	0	0	0	0
Bolzano	0.08	0	1	0	0	0	0	0	0	0	0	0	0	0	0	0	0	0	0.38	0	0
Calabria	0	0	0	1	0	0	0	0	0	0	0	0	0	0	0	0	0	0	0	0	0
Campania	0	0	0	0	1	0	0	0.33	0.9	0	0	0	0	0	0	0	0	0	0	0	0
Emilia	0	0	0	0	0	1	0	0	0	0	0	0	0	0	0	0	0	0	0	0	0.38
Friuli	0	0	0	0	0	0	1	0	0	0	0.22	0	0	0.25	0	0	0	0.68	0	0	0
Lazio	0	0	0	0	0.33	0	0	1	0	0	0	0	0	0	0	0	0	0	0	0	0
Liguria	0	0	0	0	0.9	0	0	0	1	0	0	0	0	0	0	0	0.11	0	0	0	0
Lombardia	0	0	0	0	0	0	0	0	0	1	0	0	0	0	0	0	0	0	0	0	0
Marche	0	0	0	0	0	0	0.22	0	0	0	1	0	0	0.51	0	0.17	0	0.16	0	0	0
Molise	0	0.91	0	0	0	0	0	0	0	0	0	1	0	0	0	0	0	0	0	0	0
Piemonte	0	0	0	0	0	0	0	0	0	0	0	0	1	0	0	0	0	0	0	0	0
Puglia	0	0	0	0	0	0	0.25	0	0	0	0.51	0	0	1	0	0.1	0	0.46	0	0	0
Sardegna	0	0	0	0	0	0	0	0	0	0	0	0	0	0	1	0	0	0	0	0	0
Sicilia	0	0	0	0	0	0	0	0	0	0	0.17	0	0	0.1	0	1	0	0	0	0	0
Toscana	0	0	0	0	0	0	0	0	0.11	0	0	0	0	0	0	0	1	0	0	0	0
Trento	0	0	0	0	0	0	0.68	0	0	0	0.16	0	0	0.46	0	0	0	1	0.24	0	0
Umbria	0.73	0	0.38	0	0	0	0	0	0	0	0	0	0	0	0	0	0	0.24	1	0	0
ValleAosta	0	0	0	0	0	0	0	0	0	0	0	0	0	0	0	0	0	0	0	1	0
Veneto	0	0	0	0	0	0.38	0	0	0	0	0	0	0	0	0	0	0	0	0	0	1

Table S33. Similarity Matrix of Discharged or Healed network in the third period.

	Abruzzo	Basilicata	Bolzano	Calabria	Campania	Emilia	Friuli	Lazio	Liguria	Lombardia	Marche	Molise	Piemonte	Puglia	Sardegna	Sicilia	Toscana	Trento	Umbria	ValleAosta	Veneto
Abruzzo	1	0	0	0	0	0	0	0	0	0	0	0	0	0	0	0	0	0	0	0	0
Basilicata	0	1	0	0	0	0	0	0	0	0	0	0	0	0	0	0	0	0	0	0	0
Bolzano	0	0	1	0	0	0	0	0	0	0	0	0	0	0	0	0	0	0	0	0	0
Calabria	0	0	0	1	0	0	0	0	0	0	0	0	0	0	0	0	0	0.67	0	0	0
Campania	0	0	0	0	1	0.1	0	0.17	0	0	0	0	0	0	0	0	0	0	0	0	0
Emilia	0	0	0	0	0.1	1	0	0	0	0	0	0	0	0	0	0	0	0	0	0	0
Friuli	0	0	0	0	0	0	1	0	0.27	0	0	0	0	0	0	0	0	0	0	0	0
Lazio	0	0	0	0	0.17	0	0	1	0	0	0	0	0	0	0	0	0	0	0	0	0
Liguria	0	0	0	0	0	0	0.27	0	1	0	0	0	0	0	0	0	0	0	0	0	0
Lombardia	0	0	0	0	0	0	0	0	0	1	0	0	0	0	0	0	0	0	0	0	0
Marche	0	0	0	0	0	0	0	0	0	0	1	0	0	0	0	0	0	0	0	0	0
Molise	0	0	0	0	0	0	0	0	0	0	0	1	0	0	0	0	0	0	0	0	0
Piemonte	0	0	0	0	0	0	0	0	0	0	0	0	1	0	0	0	0	0	0	0	0
Puglia	0	0	0	0	0	0	0	0	0	0	0	0	0	1	0	0.1	0	0	0	0	0
Sardegna	0	0	0	0	0	0	0	0	0	0	0	0	0	0	1	0	0	0	0	0	0
Sicilia	0	0	0	0	0	0	0	0	0	0	0	0	0	0.1	0	1	0	0	0	0	0
Toscana	0	0	0	0	0	0	0	0	0	0	0	0	0	0	0	0	1	0	0	0	0
Trento	0	0	0	0.67	0	0	0	0	0	0	0	0	0	0	0	0	0	1	0	0	0
Umbria	0	0	0	0	0	0	0	0	0	0	0	0	0	0	0	0	0	0	1	0	0
ValleAosta	0	0	0	0	0	0	0	0	0	0	0	0	0	0	0	0	0	0	0	1	0
Veneto	0	0	0	0	0	0	0	0	0	0	0	0	0	0	0	0	0	0	0	0	1

Table S34. Similarity Matrix of Discharged or Healed network in the fourth period.

	Abruzzo	Basilicata	Bolzano	Calabria	Campania	Emilia	Friuli	Lazio	Liguria	Lombardia	Marche	Molise	Piemonte	Puglia	Sardegna	Sicilia	Toscana	Trento	Umbria	ValleAosta	Veneto
Abruzzo	1	0	0	0	0	0	0	0	0	0	0	0	0	0	0	0	0	0	0	0	0
Basilicata	0	1	0	0	0	0	0	0	0	0	0	0	0	0	0	0	0	0	0	0	0
Bolzano	0	0	1	0	0	0	0	0	0	0	0	0	0	0	0	0	0	0	0	0	0
Calabria	0	0	0	1	0	0	0	0	0	0	0	0	0	0	0	0	0	0	0	0	0
Campania	0	0	0	0	1	0	0	0	0	0	0	0	0	0	0	0	0	0	0	0	0.89
Emilia	0	0	0	0	0	1	0	0	0	0	0	0	0	0	0	0	0	0	0	0	0
Friuli	0	0	0	0	0	0	1	0	0	0	0	0	0	0	0	0	0	0	0	0	0
Lazio	0	0	0	0	0	0	0	1	0	0	0	0	0	0	0	0	0	0	0	0	0
Liguria	0	0	0	0	0	0	0	0	1	0	0.36	0	0	0	0	0	0	0	0	0	0
Lombardia	0	0	0	0	0	0	0	0	0	1	0	0	0	0	0	0	0	0	0	0	0
Marche	0	0	0	0	0	0	0	0	0.36	0	1	0	0	0	0	0	0	0	0	0	0
Molise	0	0	0	0	0	0	0	0	0	0	0	1	0	0	0	0	0	0	0	0	0
Piemonte	0	0	0	0	0	0	0	0	0	0	0	0	1	0	0	0	0	0	0	0	0
Puglia	0	0	0	0	0	0	0	0	0	0	0	0	0	1	0	0	0	0	0	0	0
Sardegna	0	0	0	0	0	0	0	0	0	0	0	0	0	0	1	0	0	0	1	0	0
Sicilia	0	0	0	0	0	0	0	0	0	0	0	0	0	0	0	1	0	0	0	0	0
Toscana	0	0	0	0	0	0	0	0	0	0	0	0	0	0	0	0	1	0	0	0	0
Trento	0	0	0	0	0	0	0	0	0	0	0	0	0	0	0	0	0	1	0	0	0
Umbria	0	0	0	0	0	0	0	0	0	0	0	0	0	0	1	0	0	0	1	0	0
ValleAosta	0	0	0	0	0	0	0	0	0	0	0	0	0	0	0	0	0	0	0	1	0
Veneto	0	0	0	0	0.89	0	0	0	0	0	0	0	0	0	0	0	0	0	0	0	1

Table S35. Similarity Matrix of Discharged or Healed network in the fifth period.

	Abruzzo	Basilicata	Bolzano	Calabria	Campania	Emilia	Friuli	Lazio	Liguria	Lombardia	Marche	Molise	Piemonte	Puglia	Sardegna	Sicilia	Toscana	Trento	Umbria	ValleAosta	Veneto
Abruzzo	1	0	0	0	0	0	0	0	0	0	0	0	0	0	0	0	0	0	0	0	0
Basilicata	0	1	0	0	0	0	0	0	0	0	0	0	0	0	0	0	0	0	0	0	0
Bolzano	0	0	1	0	0	0	0	0	0	0	0	0	0	0	0	0	0	0	0	0	0
Calabria	0	0	0	1	0	0	0	0	0	0	0	0	0	0	0	0	0	0	0	0	0
Campania	0	0	0	0	1	0	0	0	0	0	0	0	0	0	0	0	0	0	0	0	0.28
Emilia	0	0	0	0	0	1	0	0	0	0	0	0	0	0	0	0	0	0	0	0	0
Friuli	0	0	0	0	0	0	1	0	0	0	0	0	0	0	0	0	0	0	0	0	0
Lazio	0	0	0	0	0	0	0	1	0	0	0	0	0	0	0	0	0	0	0	0	0
Liguria	0	0	0	0	0	0	0	0	1	0	0.05	0	0	0	0	0	0	0	0	0	0
Lombardia	0	0	0	0	0	0	0	0	0	1	0	0	0	0	0	0	0	0	0	0	0
Marche	0	0	0	0	0	0	0	0	0.05	0	1	0	0	0	0	0	0	0	0	0	0
Molise	0	0	0	0	0	0	0	0	0	0	0	1	0	0	0	0	0	0	0	0	0
Piemonte	0	0	0	0	0	0	0	0	0	0	0	0	1	0	0	0	0	0	0	0	0
Puglia	0	0	0	0	0	0	0	0	0	0	0	0	0	1	0	0	0	0	0	0	0
Sardegna	0	0	0	0	0	0	0	0	0	0	0	0	0	0	1	0	0	0	0	0	0
Sicilia	0	0	0	0	0	0	0	0	0	0	0	0	0	0	0	1	0	0	0	0	0
Toscana	0	0	0	0	0	0	0	0	0	0	0	0	0	0	0	0	1	0	0	0	0
Trento	0	0	0	0	0	0	0	0	0	0	0	0	0	0	0	0	0	1	0	0	0
Umbria	0	0	0	0	0	0	0	0	0	0	0	0	0	0	0	0	0	0	1	0	0
ValleAosta	0	0	0	0	0	0	0	0	0	0	0	0	0	0	0	0	0	0	0	1	0
Veneto	0	0	0	0	0.28	0	0	0	0	0	0	0	0	0	0	0	0	0	0	0	1

Table S36. Similarity Matrix of Deceased in the first period.

	Abruzzo	Basilicata	Bolzano	Calabria	Campania	Emilia	Friuli	Lazio	Liguria	Lombardia	Marche	Molise	Piemonte	Puglia	Sardegna	Sicilia	Toscana	Trento	Umbria	ValleAosta	Veneto
Abruzzo	1	0	0	0	0.26	0	0.19	0	0	0	0	0	0	0	0	0	0	0	0	0	0
Basilicata	0	1	0	0	0	0	0	0	0	0	0	0	0	0	0	0	0	0	0	0	0
Bolzano	0	0	1	0	0	0	0.14	0	0	0	0	0	0	0	0	0	0	0	0	0	0
Calabria	0	0	0	1	0	0	0	0	0	0	0	0	0	0	0	0	0	0	0	0	0
Campania	0.26	0	0	0	1	0	0	0	0	0	0	0	0	0.06	0	0	0	0	0	0	0
Emilia	0	0	0	0	0	1	0	0	0	0	0	0	0	0	0	0	0	0	0	0	0
Friuli	0.19	0	0.14	0	0	0	1	0	0	0	0	0	0	0	0	0	0	0	0	0	0
Lazio	0	0	0	0	0	0	0	1	0	0	0	0	0	0.14	0	0	0	0.14	0	0	0
Liguria	0	0	0	0	0	0	0	0	1	0	0	0	0	0	0	0	0	0	0	0	0
Lombardia	0	0	0	0	0	0	0	0	0	1	0	0	0	0	0	0	0	0	0	0	0
Marche	0	0	0	0	0	0	0	0	0	0	1	0	0	0	0	0	0.24	0	0	0	0
Molise	0	0	0	0	0	0	0	0	0	0	0	1	0	0	0	0	0	0	0	0	0
Piemonte	0	0	0	0	0	0	0	0	0	0	0	0	1	0	0	0	0	0	0	0	0
Puglia	0	0	0	0	0.06	0	0	0.14	0	0	0	0	0	1	0	0	0	0.63	0	0	0
Sardegna	0	0	0	0	0	0	0	0	0	0	0	0	0	0	1	0	0	0	0	0	0
Sicilia	0	0	0	0	0	0	0	0	0	0	0	0	0	0	0	1	0	0	0	0	0
Toscana	0	0	0	0	0	0	0	0	0	0	0.24	0	0	0	0	0	1	0	0	0	0
Trento	0	0	0	0	0	0	0	0.14	0	0	0	0	0	0.63	0	0	0	1	0	0	0
Umbria	0	0	0	0	0	0	0	0	0	0	0	0	0	0	0	0	0	0	1	0	0
ValleAosta	0	0	0	0	0	0	0	0	0	0	0	0	0	0	0	0	0	0	0	1	0
Veneto	0	0	0	0	0	0	0	0	0	0	0	0	0	0	0	0	0	0	0	0	1

Table S37. Similarity Matrix of Deceased in the second period.

	Abruzzo	Basilicata	Bolzano	Calabria	Campania	Emilia	Friuli	Lazio	Liguria	Lombardia	Marche	Molise	Piemonte	Puglia	Sardegna	Sicilia	Toscana	Trento	Umbria	ValleAosta	Veneto
Abruzzo	1	0	0	0	0	0	0	0	0	0	0	0	0	0	0	0.1	0	0	0	0	0
Basilicata	0	1	0	0	0	0	0	0	0	0	0	0	0	0	0	0	0	0	0	0	0
Bolzano	0	0	1	0	0	0	0	0	0	0	0	0	0	0	0	0	0	0	0	0	0
Calabria	0	0	0	1	0	0	0	0	0	0	0	0	0	0	0	0	0	0	0.15	0	0
Campania	0	0	0	0	1	0	0	0	0	0	0.39	0	0	0.73	0	0	0	0	0	0	0
Emilia	0	0	0	0	0	1	0	0	0	0	0	0	0.32	0	0	0	0	0	0	0	0
Friuli	0	0	0	0	0	0	1	0	0	0	0	0	0	0	0	0	0	0.94	0	0	0
Lazio	0	0	0	0	0	0	0	1	0	0	0	0	0	0	0	0	0	0	0	0	0
Liguria	0	0	0	0	0	0	0	0	1	0	0	0	0	0	0	0	0.27	0	0	0	0
Lombardia	0	0	0	0	0	0	0	0	0	1	0	0	0	0	0	0	0	0	0	0	0
Marche	0	0	0	0	0.39	0	0	0	0	0	1	0	0	0.19	0	0.17	0	0	0	0	0
Molise	0	0	0	0	0	0	0	0	0	0	0	1	0	0	0	0	0	0	0	0	0
Piemonte	0	0	0	0	0	0.32	0	0	0	0	0	0	1	0	0	0	0	0	0	0	0
Puglia	0	0	0	0	0.73	0	0	0	0	0	0.19	0	0	1	0	0	0	0	0	0	0
Sardegna	0	0	0	0	0	0	0	0	0	0	0	0	0	0	1	0	0	0	0	0	0
Sicilia	0.1	0	0	0	0	0	0	0	0	0	0.17	0	0	0	0	1	0	0	0	0	0
Toscana	0	0	0	0	0	0	0	0	0.27	0	0	0	0	0	0	0	1	0	0	0	0
Trento	0	0	0	0	0	0	0.94	0	0	0	0	0	0	0	0	0	0	1	0	0	0
Umbria	0	0	0	0.15	0	0	0	0	0	0	0	0	0	0	0	0	0	0	1	0.6	0
ValleAosta	0	0	0	0	0	0	0	0	0	0	0	0	0	0	0	0	0	0	0.6	1	0
Veneto	0	0	0	0	0	0	0	0	0	0	0	0	0	0	0	0	0	0	0	0	1

Table S38. Similarity Matrix of Deceased in the third period.

	Abruzzo	Basilicata	Bolzano	Calabria	Campania	Emilia	Friuli	Lazio	Liguria	Lombardia	Marche	Molise	Piemonte	Puglia	Sardegna	Sicilia	Toscana	Trento	Umbria	ValleAosta	Veneto
Abruzzo	1	0	0	0	0	0	0	0	0	0	0	0	0	0	0	0	0	0	0	0	0
Basilicata	0	1	0	0	0	0	0	0	0	0	0	0	0	0	0	0	0	0	0	0.09	0
Bolzano	0	0	1	0	0	0	0	0	0	0	0	0	0	0	0	0	0	0	0	0	0
Calabria	0	0	0	1	0	0	0	0	0	0	0	0	0	0	0	0	0	0	0	0	0
Campania	0	0	0	0	1	0	0	0	0	0	0	0	0	0	0	0	0.12	0	0	0	0
Emilia	0	0	0	0	0	1	0	0	0	0	0	0	0	0	0	0	0	0	0	0	0
Friuli	0	0	0	0	0	0	1	0	0	0	0	0	0	0	0	0	0	0	0	0	0
Lazio	0	0	0	0	0	0	0	1	0	0	0	0	0	0	0	0	0	0	0	0	0
Liguria	0	0	0	0	0	0	0	0	1	0	0	0	0	0	0	0	0	0	0	0	0
Lombardia	0	0	0	0	0	0	0	0	0	1	0	0	0	0	0	0	0	0	0	0	0
Marche	0	0	0	0	0	0	0	0	0	0	1	0	0	0	0	0	0	0	0	0	0
Molise	0	0	0	0	0	0	0	0	0	0	0	1	0	0	0	0	0	0	0	0	0
Piemonte	0	0	0	0	0	0	0	0	0	0	0	0	1	0	0	0	0	0	0	0	0.36
Puglia	0	0	0	0	0	0	0	0	0	0	0	0	0	1	0	0.51	0	0	0	0	0
Sardegna	0	0	0	0	0	0	0	0	0	0	0	0	0	0	1	0	0	0.07	0	0	0
Sicilia	0	0	0	0	0	0	0	0	0	0	0	0	0	0.51	0	1	0	0	0	0	0
Toscana	0	0	0	0	0.12	0	0	0	0	0	0	0	0	0	0	0	1	0	0	0	0
Trento	0	0	0	0	0	0	0	0	0	0	0	0	0	0	0.07	0	0	1	0	0	0
Umbria	0	0	0	0	0	0	0	0	0	0	0	0	0	0	0	0	0	0	1	0	0
ValleAosta	0	0.09	0	0	0	0	0	0	0	0	0	0	0	0	0	0	0	0	0	1	0
Veneto	0	0	0	0	0	0	0	0	0	0	0	0	0.36	0	0	0	0	0	0	0	1

Table S39. Similarity Matrix of Deceased in the fourth period.

	Abruzzo	Basilicata	Bolzano	Calabria	Campania	Emilia	Friuli	Lazio	Liguria	Lombardia	Marche	Molise	Piemonte	Puglia	Sardegna	Sicilia	Toscana	Trento	Umbria	ValleAosta	Veneto
Abruzzo	1	0	0	0	0	0	0	0	0	0	0	0	0	0	0	0	0	0	0	0	0
Basilicata	0	1	0	0	0	0	0	0	0	0	0	0	0	0	0	0	0	0	0	0	0
Bolzano	0	0	1	0	0	0	0	0	0	0	0	0	0	0	0	0	0	0	0	0	0
Calabria	0	0	0	1	0	0	0	0	0	0	0	0	0	0	0	0	0	0	0	0	0
Campania	0	0	0	0	1	0	0	0	0	0	0	0	0	0	0	0	0	0	0	0	0
Emilia	0	0	0	0	0	1	0	0	0	0	0	0	0	0	0	0	0	0	0	0	0
Friuli	0	0	0	0	0	0	1	0	0	0	0	0	0	0	0	0	0	0	0	0	0
Lazio	0	0	0	0	0	0	0	1	0	0	0	0	0	0	0	0	0	0	0	0	0
Liguria	0	0	0	0	0	0	0	0	1	0	0	0	0	0	0	0	0	0	0	0	0
Lombardia	0	0	0	0	0	0	0	0	0	1	0	0	0	0	0	0	0	0	0	0	0
Marche	0	0	0	0	0	0	0	0	0	0	1	0	0	0	0	0	0	0	0	0	0
Molise	0	0	0	0	0	0	0	0	0	0	0	1	0	0	0	0	0	0	0	0	0
Piemonte	0	0	0	0	0	0	0	0	0	0	0	0	1	0	0	0	0	0	0	0	0
Puglia	0	0	0	0	0	0	0	0	0	0	0	0	0	1	0	0	0	0	0	0	0
Sardegna	0	0	0	0	0	0	0	0	0	0	0	0	0	0	1	0	0	0	0	0	0
Sicilia	0	0	0	0	0	0	0	0	0	0	0	0	0	0	0	1	0	0	0	0	0
Toscana	0	0	0	0	0	0	0	0	0	0	0	0	0	0	0	0	1	0	0	0	0
Trento	0	0	0	0	0	0	0	0	0	0	0	0	0	0	0	0	0	1	0	0	0
Umbria	0	0	0	0	0	0	0	0	0	0	0	0	0	0	0	0	0	0	1	0	0
ValleAosta	0	0	0	0	0	0	0	0	0	0	0	0	0	0	0	0	0	0	0	1	0
Veneto	0	0	0	0	0	0	0	0	0	0	0	0	0	0	0	0	0	0	0	0	1

Table S40. Similarity Matrix of Deceased in the fifth period.

	Abruzzo	Basilicata	Bolzano	Calabria	Campania	Emilia	Friuli	Lazio	Liguria	Lombardia	Marche	Molise	Piemonte	Puglia	Sardegna	Sicilia	Toscana	Trento	Umbria	ValleAosta	Veneto
Abruzzo	1	0	0	0	0	0	0	0	0	0	0	0	0	0	0	0	0	0	0	0	0
Basilicata	0	1	0	0	0	0	0	0	0	0	0	0	0	0	0	0	0	0	0	0	0
Bolzano	0	0	1	0	0	0	0	0	0	0	0	0	0	0	0	0	0	0	0	0	0
Calabria	0	0	0	1	0	0	0	0	0	0	0	0	0	0	0	0	0	0	0	0	0
Campania	0	0	0	0	1	0	0	0	0	0	0	0	0	0	0	0	0	0	0	0	0
Emilia	0	0	0	0	0	1	0	0	0	0	0	0	0	0	0	0	0	0	0	0	0
Friuli	0	0	0	0	0	0	1	0	0	0	0	0	0	0	0	0	0	0	0	0	0
Lazio	0	0	0	0	0	0	0	1	0	0	0	0	0	0	0	0	0	0	0	0	0
Liguria	0	0	0	0	0	0	0	0	1	0	0	0	0	0	0	0	0	0	0	0	0
Lombardia	0	0	0	0	0	0	0	0	0	1	0	0	0	0	0	0	0	0	0	0	0
Marche	0	0	0	0	0	0	0	0	0	0	1	0	0	0	0	0	0	0	0	0	0
Molise	0	0	0	0	0	0	0	0	0	0	0	1	0	0	0	0	0	0	0	0	0
Piemonte	0	0	0	0	0	0	0	0	0	0	0	0	1	0	0	0	0	0	0	0	0
Puglia	0	0	0	0	0	0	0	0	0	0	0	0	0	1	0	0	0	0	0	0	0
Sardegna	0	0	0	0	0	0	0	0	0	0	0	0	0	0	1	0	0	0	0	0	0
Sicilia	0	0	0	0	0	0	0	0	0	0	0	0	0	0	0	1	0	0	0	0	0
Toscana	0	0	0	0	0	0	0	0	0	0	0	0	0	0	0	0	1	0	0	0	0
Trento	0	0	0	0	0	0	0	0	0	0	0	0	0	0	0	0	0	1	0	0	0
Umbria	0	0	0	0	0	0	0	0	0	0	0	0	0	0	0	0	0	0	1	0	0
ValleAosta	0	0	0	0	0	0	0	0	0	0	0	0	0	0	0	0	0	0	0	1	0
Veneto	0	0	0	0	0	0	0	0	0	0	0	0	0	0	0	0	0	0	0	0	1

Table S41. Similarity Matrix of Total Cases in the first period.

	Abruzzo	Basilicata	Bolzano	Calabria	Campania	Emilia	Friuli	Lazio	Liguria	Lombardia	Marche	Molise	Piemonte	Puglia	Sardegna	Sicilia	Toscana	Trento	Umbria	ValleAosta	Veneto
Abruzzo	1	0	0	0	0	0	0.42	0	0	0	0	0	0	0	0	0.07	0	0	0	0	0
Basilicata	0	1	0	0	0	0	0	0	0	0	0	0.34	0	0	0	0	0	0	0	0	0
Bolzano	0	0	1	0	0	0	0	0	0	0	0	0	0	0	0	0	0	0	0	0	0
Calabria	0	0	0	1	0	0	0	0	0	0	0	0	0	0	0	0	0	0	0	0.36	0
Campania	0	0	0	0	1	0	0	0	0	0	0	0	0	0	0	0	0	0	0	0	0
Emilia	0	0	0	0	0	1	0	0	0	0	0	0	0.82	0	0	0	0	0	0	0	0
Friuli	0.42	0	0	0	0	0	1	0	0	0	0	0	0	0	0	0.25	0	0	0	0	0
Lazio	0	0	0	0	0	0	0	1	0	0	0.42	0	0	0	0	0	0	0	0	0	0
Liguria	0	0	0	0	0	0	0	0	1	0	0	0	0	0	0	0	0	0	0	0	0
Lombardia	0	0	0	0	0	0	0	0	0	1	0	0	0	0	0	0	0	0	0	0	0
Marche	0	0	0	0	0	0	0	0.42	0	0	1	0	0	0	0	0	0	0	0	0	0
Molise	0	0.34	0	0	0	0	0	0	0	0	0	1	0	0	0	0	0	0	0	0	0
Piemonte	0	0	0	0	0	0.82	0	0	0	0	0	0	1	0	0	0	0	0	0	0	0
Puglia	0	0	0	0	0	0	0	0	0	0	0	0	0	1	0	0	0	0.8	0	0	0
Sardegna	0	0	0	0	0	0	0	0	0	0	0	0	0	0	1	0	0	0	0	0	0
Sicilia	0.07	0	0	0	0	0	0.25	0	0	0	0	0	0	0	0	1	0	0	0	0	0
Toscana	0	0	0	0	0	0	0	0	0	0	0	0	0	0	0	0	1	0	0	0	0
Trento	0	0	0	0	0	0	0	0	0	0	0	0	0	0.8	0	0	0	1	0	0	0
Umbria	0	0	0	0	0	0	0	0	0	0	0	0	0	0	0	0	0	0	1	0	0
ValleAosta	0	0	0	0.36	0	0	0	0	0	0	0	0	0	0	0	0	0	0	0	1	0
Veneto	0	0	0	0	0	0	0	0	0	0	0	0	0	0	0	0	0	0	0	0	1

Table S42. Similarity Matrix of Total Cases in the second period.

	Abruzzo	Basilicata	Bolzano	Calabria	Campania	Emilia	Friuli	Lazio	Liguria	Lombardia	Marche	Molise	Piemonte	Puglia	Sardegna	Sicilia	Toscana	Trento	Umbria	ValleAosta	Veneto
Abruzzo	1	0	0	0	0	0	0.16	0	0	0	0	0	0	0	0	0	0	0	0	0	0
Basilicata	0	1	0	0	0	0	0	0	0	0	0	0	0	0	0	0	0	0	0	0.34	0
Bolzano	0	0	1	0	0	0	0	0	0	0	0	0	0	0	0.69	0	0	0	0.86	0	0
Calabria	0	0	0	1	0	0	0	0	0	0	0	0	0	0	0	0	0	0.07	0	0	0
Campania	0	0	0	0	1	0.24	0	0	0	0	0	0	0	0	0	0	0	0	0	0	0.31
Emilia	0	0	0	0	0.24	1	0	0.12	0	0	0	0	0	0	0	0	0	0	0	0	0.3
Friuli	0.16	0	0	0	0	0	1	0	0	0	0.48	0	0	0	0	0	0	0	0	0	0
Lazio	0	0	0	0	0	0.12	0	1	0	0	0	0	0	0	0	0	0	0	0	0	0
Liguria	0	0	0	0	0	0	0	0	1	0	0	0	0	0.8	0	0.28	0	0	0	0	0
Lombardia	0	0	0	0	0	0	0	0	0	1	0	0	0	0	0	0	0	0	0	0	0
Marche	0	0	0	0	0	0	0.48	0	0	0	1	0	0	0	0	0	0	0	0	0	0
Molise	0	0	0	0	0	0	0	0	0	0	0	1	0	0	0	0	0	0	0	0	0
Piemonte	0	0	0	0	0	0	0	0	0	0	0	0	1	0	0	0	0	0	0	0	0.28
Puglia	0	0	0	0	0	0	0	0	0.8	0	0	0	0	1	0	0.38	0	0	0	0	0
Sardegna	0	0	0.69	0	0	0	0	0	0	0	0	0	0	0	1	0	0	0	0.85	0	0
Sicilia	0	0	0	0	0	0	0	0	0.28	0	0	0	0	0.38	0	1	0	0	0	0	0
Toscana	0	0	0	0	0	0	0	0	0	0	0	0	0	0	0	0	1	0	0	0	0
Trento	0	0	0	0.07	0	0	0	0	0	0	0	0	0	0	0	0	0	1	0	0	0
Umbria	0	0	0.86	0	0	0	0	0	0	0	0	0	0	0	0.85	0	0	0	1	0	0
ValleAosta	0	0.34	0	0	0	0	0	0	0	0	0	0	0	0	0	0	0	0	0	1	0
Veneto	0	0	0	0	0.31	0.3	0	0	0	0	0	0	0.28	0	0	0	0	0	0	0	1

Table S43. Similarity Matrix of Total Cases in the third period.

	Abruzzo	Basilicata	Bolzano	Calabria	Campania	Emilia	Friuli	Lazio	Liguria	Lombardia	Marche	Molise	Piemonte	Puglia	Sardegna	Sicilia	Toscana	Trento	Umbria	ValleAosta	Veneto
Abruzzo	1	0	0.56	0	0	0	0	0	0	0	0	0	0	0	0	0	0	0	0	0	0
Basilicata	0	1	0	0	0	0	0	0	0	0	0	0	0	0	0	0	0	0	0	0	0
Bolzano	0.56	0	1	0	0	0	0	0	0	0	0	0	0	0	0	0	0	0	0	0	0
Calabria	0	0	0	1	0	0	0	0	0	0	0	0	0	0	0.32	0	0	0	0.23	0	0
Campania	0	0	0	0	1	0.11	0	0	0	0	0	0	0	0	0	0	0	0	0	0	0
Emilia	0	0	0	0	0.11	1	0	0	0	0	0	0	0.05	0	0	0	0	0	0	0	0
Friuli	0	0	0	0	0	0	1	0	0.09	0	0	0	0	0	0	0	0	0	0	0	0
Lazio	0	0	0	0	0	0	0	1	0	0	0	0	0	0	0	0	0	0	0	0	0
Liguria	0	0	0	0	0	0	0.09	0	1	0	0	0	0	0	0	0	0	0	0	0	0
Lombardia	0	0	0	0	0	0	0	0	0	1	0	0	0	0	0	0	0	0	0	0	0
Marche	0	0	0	0	0	0	0	0	0	0	1	0	0	0	0	0	0	0	0	0	0
Molise	0	0	0	0	0	0	0	0	0	0	0	1	0	0	0	0	0	0	0	0	0
Piemonte	0	0	0	0	0	0.05	0	0	0	0	0	0	1	0	0	0	0	0	0	0	0
Puglia	0	0	0	0	0	0	0	0	0	0	0	0	0	1	0	0.25	0.5	0	0	0	0
Sardegna	0	0	0	0.32	0	0	0	0	0	0	0	0	0	0	1	0	0	0	0.35	0	0
Sicilia	0	0	0	0	0	0	0	0	0	0	0	0	0	0.25	0	1	0	0	0	0	0
Toscana	0	0	0	0	0	0	0	0	0	0	0	0	0	0.5	0	0	1	0	0	0	0
Trento	0	0	0	0	0	0	0	0	0	0	0	0	0	0	0	0	0	1	0	0	0
Umbria	0	0	0	0.23	0	0	0	0	0	0	0	0	0	0	0.35	0	0	0	1	0	0
ValleAosta	0	0	0	0	0	0	0	0	0	0	0	0	0	0	0	0	0	0	0	1	0
Veneto	0	0	0	0	0	0	0	0	0	0	0	0	0	0	0	0	0	0	0	0	1

Table S44. Similarity Matrix of Total Cases in the fourth period.

	Abruzzo	Basilicata	Bolzano	Calabria	Campania	Emilia	Friuli	Lazio	Liguria	Lombardia	Marche	Molise	Piemonte	Puglia	Sardegna	Sicilia	Toscana	Trento	Umbria	ValleAosta	Veneto
Abruzzo	1	0	0	0	0	0	0	0	0	0	0	0	0	0	0	0	0	0	0	0	0
Basilicata	0	1	0	0	0	0	0	0	0	0	0	0	0	0	0	0	0	0	0	0	0
Bolzano	0	0	1	0	0	0	0	0	0	0	0	0	0	0	0	0	0	0	0	0	0
Calabria	0	0	0	1	0	0	0	0	0	0	0	0	0	0	0	0	0	0	0	0	0
Campania	0	0	0	0	1	0	0	0	0	0	0	0	0	0	0	0	0	0	0	0	0
Emilia	0	0	0	0	0	1	0	0	0	0	0	0	0	0	0	0	0	0	0	0	0
Friuli	0	0	0	0	0	0	1	0	0	0	0	0	0	0	0	0	0	0	0	0	0
Lazio	0	0	0	0	0	0	0	1	0	0	0	0	0.19	0	0	0	0	0	0	0	0
Liguria	0	0	0	0	0	0	0	0	1	0	0.1	0	0	0	0	0	0	0	0	0	0
Lombardia	0	0	0	0	0	0	0	0	0	1	0	0	0	0	0	0	0	0	0	0	0
Marche	0	0	0	0	0	0	0	0	0.1	0	1	0	0	0	0	0	0	0	0	0	0
Molise	0	0	0	0	0	0	0	0	0	0	0	1	0	0	0	0	0	0	0	0	0
Piemonte	0	0	0	0	0	0	0	0.19	0	0	0	0	1	0	0	0	0	0	0	0	0
Puglia	0	0	0	0	0	0	0	0	0	0	0	0	0	1	0	0	0	0	0	0	0
Sardegna	0	0	0	0	0	0	0	0	0	0	0	0	0	0	1	0	0	0	0	0	0
Sicilia	0	0	0	0	0	0	0	0	0	0	0	0	0	0	0	1	0	0	0	0	0
Toscana	0	0	0	0	0	0	0	0	0	0	0	0	0	0	0	0	1	0	0	0	0
Trento	0	0	0	0	0	0	0	0	0	0	0	0	0	0	0	0	0	1	0	0	0
Umbria	0	0	0	0	0	0	0	0	0	0	0	0	0	0	0	0	0	0	1	0	0
ValleAosta	0	0	0	0	0	0	0	0	0	0	0	0	0	0	0	0	0	0	0	1	0
Veneto	0	0	0	0	0	0	0	0	0	0	0	0	0	0	0	0	0	0	0	0	1

Table S45. Similarity Matrix of Total Cases in the fifth period.

	Abruzzo	Basilicata	Bolzano	Calabria	Campania	Emilia	Friuli	Lazio	Liguria	Lombardia	Marche	Molise	Piemonte	Puglia	Sardegna	Sicilia	Toscana	Trento	Umbria	ValleAosta	Veneto
Abruzzo	1	0	0	0	0	0	0	0	0	0	0	0	0	0	0	0	0	0	0	0	0
Basilicata	0	1	0	0	0	0	0	0	0	0	0	0	0	0	0	0	0	0	0	0	0
Bolzano	0	0	1	0	0	0	0	0	0	0	0	0	0	0	0	0	0	0	0	0	0
Calabria	0	0	0	1	0	0	0	0	0	0	0	0	0	0	0	0	0	0	0	0	0
Campania	0	0	0	0	1	0	0	0	0	0	0	0	0	0	0	0	0	0	0	0	0.94
Emilia	0	0	0	0	0	1	0	0	0	0	0	0	0.08	0	0	0	0	0	0	0	0
Friuli	0	0	0	0	0	0	1	0	0	0	0	0	0	0	0	0	0	0	0	0	0
Lazio	0	0	0	0	0	0	0	1	0	0	0	0	0	0	0	0	0	0	0	0	0
Liguria	0	0	0	0	0	0	0	0	1	0	0	0	0	0	0	0	0	0	0	0	0
Lombardia	0	0	0	0	0	0	0	0	0	1	0	0	0	0	0	0	0	0	0	0	0
Marche	0	0	0	0	0	0	0	0	0	0	1	0	0	0	0	0	0	0	0	0	0
Molise	0	0	0	0	0	0	0	0	0	0	0	1	0	0	0	0	0	0	0	0	0
Piemonte	0	0	0	0	0	0.08	0	0	0	0	0	0	1	0	0	0	0	0	0	0	0
Puglia	0	0	0	0	0	0	0	0	0	0	0	0	0	1	0	0	0	0	0	0	0
Sardegna	0	0	0	0	0	0	0	0	0	0	0	0	0	0	1	0	0	0	0	0	0
Sicilia	0	0	0	0	0	0	0	0	0	0	0	0	0	0	0	1	0	0	0	0	0
Toscana	0	0	0	0	0	0	0	0	0	0	0	0	0	0	0	0	1	0	0	0	0
Trento	0	0	0	0	0	0	0	0	0	0	0	0	0	0	0	0	0	1	0	0	0
Umbria	0	0	0	0	0	0	0	0	0	0	0	0	0	0	0	0	0	0	1	0	0
ValleAosta	0	0	0	0	0	0	0	0	0	0	0	0	0	0	0	0	0	0	0	1	0
Veneto	0	0	0	0	0.94	0	0	0	0	0	0	0	0	0	0	0	0	0	0	0	1

Table S46. Similarity Matrix of Swab in the first period.

	Abruzzo	Basilicata	Bolzano	Calabria	Campania	Emilia	Friuli	Lazio	Liguria	Lombardia	Marche	Molise	Piemonte	Puglia	Sardegna	Sicilia	Toscana	Trento	Umbria	ValleAosta	Veneto
Abruzzo	1	0	0.99	0.69	0	0	0	0	0.14	0	0	0	0	0	0	0	0	0.91	0.71	0	0
Basilicata	0	1	0	0	0	0	0	0	0	0	0	0	0	0	0	0	0	0	0	0.08	0
Bolzano	0.99	0	1	0.7	0	0	0	0	0.16	0	0	0	0	0	0	0	0	0.98	0.7	0	0
Calabria	0.69	0	0.7	1	0	0	0	0	0.08	0	0	0	0	0	0.07	0	0	0.63	0.97	0	0
Campania	0	0	0	0	1	0	0.47	0	0	0	0.16	0	0	0.2	0	0.65	0	0	0	0	0
Emilia	0	0	0	0	0	1	0	0.05	0	0	0	0	0.16	0	0	0	0.06	0	0	0	0
Friuli	0	0	0	0	0.47	0	1	0	0.09	0	0.26	0	0	0.5	0	0.63	0	0	0	0	0
Lazio	0	0	0	0	0	0.05	0	1	0	0	0	0	0.73	0	0	0	0.97	0	0	0	0
Liguria	0.14	0	0.16	0.08	0	0	0.09	0	1	0	0.5	0	0	0.26	0	0	0	0.2	0.08	0	0
Lombardia	0	0	0	0	0	0	0	0	0	1	0	0	0	0	0	0	0	0	0	0	0.55
Marche	0	0	0	0	0.16	0	0.26	0	0.5	0	1	0	0	0.69	0	0.15	0	0	0	0	0
Molise	0	0	0	0	0	0	0	0	0	0	0	1	0	0	0	0	0	0	0	0.38	0
Piemonte	0	0	0	0	0	0.16	0	0.73	0	0	0	0	1	0	0	0	0.71	0	0	0	0
Puglia	0	0	0	0	0.2	0	0.5	0	0.26	0	0.69	0	0	1	0	0.24	0	0	0	0	0
Sardegna	0	0	0	0.07	0	0	0	0	0	0	0	0	0	0	1	0	0	0.06	0.07	0	0
Sicilia	0	0	0	0	0.65	0	0.63	0	0	0	0.15	0	0	0.24	0	1	0	0	0	0	0
Toscana	0	0	0	0	0	0.06	0	0.97	0	0	0	0	0.71	0	0	0	1	0	0	0	0
Trento	0.91	0	0.98	0.63	0	0	0	0	0.2	0	0	0	0	0	0.06	0	0	1	0.66	0	0
Umbria	0.71	0	0.7	0.97	0	0	0	0	0.08	0	0	0	0	0	0.07	0	0	0.66	1	0	0
ValleAosta	0	0.08	0	0	0	0	0	0	0	0	0	0.38	0	0	0	0	0	0	0	1	0
Veneto	0	0	0	0	0	0	0	0	0	0.55	0	0	0	0	0	0	0	0	0	0	1

Table S47. Similarity Matrix of Swab in the second period.

	Abruzzo	Basilicata	Bolzano	Calabria	Campania	Emilia	Friuli	Lazio	Liguria	Lombardia	Marche	Molise	Piemonte	Puglia	Sardegna	Sicilia	Toscana	Trento	Umbria	ValleAosta	Veneto
Abruzzo	1	0	0	0	0	0	0	0	0	0	0.13	0	0	0	0.08	0	0	0.35	0.69	0	0
Basilicata	0	1	0	0	0	0	0	0	0	0	0	0	0	0	0	0	0	0	0	0	0
Bolzano	0	0	1	0	0	0	0	0	0	0	0	0	0	0	0	0	0	0	0	0	0
Calabria	0	0	0	1	0	0	0	0	0	0	0	0	0	0	0.66	0	0	0.1	0	0	0
Campania	0	0	0	0	1	0	0	0	0	0	0	0	0.85	0	0	0	0.49	0	0	0	0
Emilia	0	0	0	0	0	1	0	0.71	0	0	0	0	0	0	0	0	0	0	0	0	0
Friuli	0	0	0	0	0	0	1	0	0	0	0	0	0	0.12	0	0	0	0	0	0	0
Lazio	0	0	0	0	0	0.71	0	1	0	0	0	0	0	0	0	0	0	0	0	0	0
Liguria	0	0	0	0	0	0	0	0	1	0	0	0	0	0	0	0	0	0	0	0	0
Lombardia	0	0	0	0	0	0	0	0	0	1	0	0	0	0	0	0	0	0	0	0	0
Marche	0.13	0	0	0	0	0	0	0	0	0	1	0	0	0	0	0	0	0	0.3	0	0
Molise	0	0	0	0	0	0	0	0	0	0	0	1	0	0	0	0	0	0	0	0	0
Piemonte	0	0	0	0	0.85	0	0	0	0	0	0	0	1	0	0	0	0.19	0	0	0	0
Puglia	0	0	0	0	0	0	0.12	0	0	0	0	0	0	1	0	0	0	0	0	0	0
Sardegna	0.08	0	0	0.66	0	0	0	0	0	0	0	0	0	0	1	0	0	0.38	0	0	0
Sicilia	0	0	0	0	0	0	0	0	0	0	0	0	0	0	0	1	0	0	0	0	0
Toscana	0	0	0	0	0.49	0	0	0	0	0	0	0	0.19	0	0	0	1	0	0	0	0
Trento	0.35	0	0	0.1	0	0	0	0	0	0	0	0	0	0	0.38	0	0	1	0.1	0	0
Umbria	0.69	0	0	0	0	0	0	0	0	0	0.3	0	0	0	0	0	0	0.1	1	0	0
ValleAosta	0	0	0	0	0	0	0	0	0	0	0	0	0	0	0	0	0	0	0	1	0
Veneto	0	0	0	0	0	0	0	0	0	0	0	0	0	0	0	0	0	0	0	0	1

Table S48. Similarity Matrix of Swab in the third period.

	Abruzzo	Basilicata	Bolzano	Calabria	Campania	Emilia	Friuli	Lazio	Liguria	Lombardia	Marche	Molise	Piemonte	Puglia	Sardegna	Sicilia	Toscana	Trento	Umbria	ValleAosta	Veneto
Abruzzo	1	0	0	0	0	0	0	0	0.12	0	0	0	0	0	0	0	0	0	0	0	0
Basilicata	0	1	0	0	0	0	0	0	0	0	0	0	0	0	0	0	0	0	0	0	0
Bolzano	0	0	1	0	0	0	0	0	0	0	0.41	0	0	0	0.26	0	0	0	0.41	0	0
Calabria	0	0	0	1	0	0	0	0	0	0	0	0	0	0	0	0	0	0.4	0	0	0
Campania	0	0	0	0	1	0	0	0	0	0	0	0	0	0	0	0	0.07	0	0	0	0
Emilia	0	0	0	0	0	1	0	0	0	0	0	0	0	0	0	0	0	0	0	0	0
Friuli	0	0	0	0	0	0	1	0	0	0	0	0	0	0	0	0	0	0	0	0	0
Lazio	0	0	0	0	0	0	0	1	0	0	0	0	0	0	0	0	0	0	0	0	0
Liguria	0.12	0	0	0	0	0	0	0	1	0	0	0	0	0	0	0	0	0	0	0	0
Lombardia	0	0	0	0	0	0	0	0	0	1	0	0	0	0	0	0	0	0	0	0	0
Marche	0	0	0.41	0	0	0	0	0	0	0	1	0	0	0	0.61	0	0	0	0.87	0	0
Molise	0	0	0	0	0	0	0	0	0	0	0	1	0	0	0	0	0	0	0	0	0
Piemonte	0	0	0	0	0	0	0	0	0	0	0	0	1	0	0	0.13	0.09	0	0	0	0
Puglia	0	0	0	0	0	0	0	0	0	0	0	0	0	1	0	0	0	0	0	0	0
Sardegna	0	0	0.26	0	0	0	0	0	0	0	0.61	0	0	0	1	0	0	0	0.53	0	0
Sicilia	0	0	0	0	0	0	0	0	0	0	0	0	0.13	0	0	1	0	0	0	0	0
Toscana	0	0	0	0	0.07	0	0	0	0	0	0	0	0.09	0	0	0	1	0	0	0	0
Trento	0	0	0	0.4	0	0	0	0	0	0	0	0	0	0	0	0	0	1	0	0	0
Umbria	0	0	0.41	0	0	0	0	0	0	0	0.87	0	0	0	0.53	0	0	0	1	0	0
ValleAosta	0	0	0	0	0	0	0	0	0	0	0	0	0	0	0	0	0	0	0	1	0
Veneto	0	0	0	0	0	0	0	0	0	0	0	0	0	0	0	0	0	0	0	0	1

Table S49. Similarity Matrix of Swab in the fourth period.

	Abruzzo	Basilicata	Bolzano	Calabria	Campania	Emilia	Friuli	Lazio	Liguria	Lombardia	Marche	Molise	Piemonte	Puglia	Sardegna	Sicilia	Toscana	Trento	Umbria	ValleAosta	Veneto
Abruzzo	1	0	0.29	0	0	0	0	0	0	0	0	0	0	0	0	0	0	0	0	0	0
Basilicata	0	1	0	0	0	0	0	0	0	0	0	0	0	0	0	0	0	0	0	0	0
Bolzano	0.29	0	1	0	0	0	0	0	0	0	0	0	0	0	0	0	0	0	0	0	0
Calabria	0	0	0	1	0	0	0	0	0	0	0	0	0	0	0	0	0	0	0	0	0
Campania	0	0	0	0	1	0	0	0	0	0	0	0	0.9	0	0	0	0.1	0	0	0	0
Emilia	0	0	0	0	0	1	0	0	0	0	0	0	0	0	0	0	0	0	0	0	0
Friuli	0	0	0	0	0	0	1	0	0	0	0	0	0	0	0	0	0	0	0	0	0
Lazio	0	0	0	0	0	0	0	1	0	0	0	0	0	0	0	0	0	0	0	0	0
Liguria	0	0	0	0	0	0	0	0	1	0	0	0	0	0	0	0	0	0	0	0	0
Lombardia	0	0	0	0	0	0	0	0	0	1	0	0	0	0	0	0	0	0	0	0	0
Marche	0	0	0	0	0	0	0	0	0	0	1	0	0	0	0	0	0	0	0	0	0
Molise	0	0	0	0	0	0	0	0	0	0	0	1	0	0	0	0	0	0	0	0	0
Piemonte	0	0	0	0	0.9	0	0	0	0	0	0	0	1	0	0	0	0.37	0	0	0	0
Puglia	0	0	0	0	0	0	0	0	0	0	0	0	0	1	0	0	0	0	0	0	0
Sardegna	0	0	0	0	0	0	0	0	0	0	0	0	0	0	1	0	0	0	0	0	0
Sicilia	0	0	0	0	0	0	0	0	0	0	0	0	0	0	0	1	0	0	0	0	0
Toscana	0	0	0	0	0.1	0	0	0	0	0	0	0	0.37	0	0	0	1	0	0	0	0
Trento	0	0	0	0	0	0	0	0	0	0	0	0	0	0	0	0	0	1	0	0	0
Umbria	0	0	0	0	0	0	0	0	0	0	0	0	0	0	0	0	0	0	1	0	0
ValleAosta	0	0	0	0	0	0	0	0	0	0	0	0	0	0	0	0	0	0	0	1	0
Veneto	0	0	0	0	0	0	0	0	0	0	0	0	0	0	0	0	0	0	0	0	1

Table S50. Similarity Matrix of Swab in the fifth period.

	Abruzzo	Basilicata	Bolzano	Calabria	Campania	Emilia	Friuli	Lazio	Liguria	Lombardia	Marche	Molise	Piemonte	Puglia	Sardegna	Sicilia	Toscana	Trento	Umbria	ValleAosta	Veneto
Abruzzo	1	0	0	0	0	0	0	0	0	0	0	0	0	0	0	0	0	0	0	0	0
Basilicata	0	1	0	0	0	0	0	0	0	0	0	0	0	0	0	0	0	0	0	0	0
Bolzano	0	0	1	0	0	0	0	0	0	0	0	0	0	0	0	0	0	0	0	0	0
Calabria	0	0	0	1	0	0	0	0	0	0	0	0	0	0	0	0	0	0	0	0	0
Campania	0	0	0	0	1	0	0	0	0	0	0	0	0	0	0	0	0.16	0	0	0	0
Emilia	0	0	0	0	0	1	0	0	0	0	0	0	0.3	0	0	0	0	0	0	0	0
Friuli	0	0	0	0	0	0	1	0	0	0	0	0	0	0	0	0	0	0	0	0	0
Lazio	0	0	0	0	0	0	0	1	0	0	0	0	0	0	0	0	0	0	0	0	0.42
Liguria	0	0	0	0	0	0	0	0	1	0	0	0	0	0	0	0	0	0	0	0	0
Lombardia	0	0	0	0	0	0	0	0	0	1	0	0	0	0	0	0	0	0	0	0	0
Marche	0	0	0	0	0	0	0	0	0	0	1	0	0	0	0	0	0	0	0	0	0
Molise	0	0	0	0	0	0	0	0	0	0	0	1	0	0	0	0	0	0	0	0	0
Piemonte	0	0	0	0	0	0.3	0	0	0	0	0	0	1	0	0	0	0	0	0	0	0
Puglia	0	0	0	0	0	0	0	0	0	0	0	0	0	1	0	0	0	0	0	0	0
Sardegna	0	0	0	0	0	0	0	0	0	0	0	0	0	0	1	0	0	0	0.29	0	0
Sicilia	0	0	0	0	0	0	0	0	0	0	0	0	0	0	0	1	0	0	0	0	0
Toscana	0	0	0	0	0.16	0	0	0	0	0	0	0	0	0	0	0	1	0	0	0	0
Trento	0	0	0	0	0	0	0	0	0	0	0	0	0	0	0	0	0	1	0	0	0
Umbria	0	0	0	0	0	0	0	0	0	0	0	0	0	0	0.29	0	0	0	1	0	0
ValleAosta	0	0	0	0	0	0	0	0	0	0	0	0	0	0	0	0	0	0	0	1	0
Veneto	0	0	0	0	0	0	0	0.42	0	0	0	0	0	0	0	0	0	0	0	0	1
